# Molecular evaluation of residual disease following neoadjuvant chemotherapy in triple-negative breast cancer CALGB 40603 (Alliance)

**DOI:** 10.1172/JCI205962

**Published:** 2026-08-04

**Authors:** Patrick D. Rädler, Brooke M. Felsheim, Aranzazu Fernandez-Martinez, Adam D. Pfefferle, Michele C. Hayward, Baljit Singh, William Sikov, Lisa A. Carey, Charles M. Perou

**Affiliations:** 1 Lineberger Comprehensive Cancer Center (LCCC) and; 2 Bioinformatics and Computational Biology Curriculum, University of North Carolina at Chapel Hill (UNC), Chapel Hill, North Carolina, USA.; 3 Drug Development Department (DITEP), INSERM UMR981, IHU-National PRecISion Medicine Center in Oncology, Gustave Roussy Cancer Campus, Villejuif, France.; 4 Department of Genetics, UNC, Chapel Hill, North Carolina, USA.; 5 White Plains Hospital, White Plains, New York, USA.; 6 Program in Women’s Oncology, Women and Infants Hospital of Rhode Island, Warren Alpert Medical School of Brown University, Providence, Rhode Island, USA.; 7 Division of Hematology-Oncology, Department of Medicine, School of Medicine, UNC, Chapel Hill, North Carolina, USA.

**Keywords:** Clinical Research, Oncology, Biomarkers, Breast cancer, Transcriptomics

## Abstract

**BACKGROUND:**

Despite therapeutic advances in early-stage triple-negative breast cancer (TNBC), residual disease (RD) following neoadjuvant therapy remains a key predictor of a worse prognosis and obstacle to improving patient outcomes.

**METHODS:**

To better characterize RD and identify survival-associated features, we performed comprehensive transcriptomic profiling of 340 pretreatment stage II/III TNBCs and 70 matched posttreatment RD samples from the randomized CALGB 40603 (Alliance) phase II clinical trial. To explore preclinical treatment strategies for RD, patient-derived xenograft (PDX) mouse models mimicking RD were treated with antibody-drug conjugates (ADCs).

**RESULTS:**

Our study shows prognostic genomic features measured pretreatment may differ from prognostic features measured posttreatment from RD specimens. Patients with a genomic PAM50 subtype of basal-like in RD specimens had a poor survival outcome, and their matching pretreatment tumors were characterized by elevated chromosomal amplifications of oncogenic drivers and significantly reduced B and T cell expression features. Paired analyses of basal-like RD and matched pretreatment tumors revealed further lymphocyte depletion in RD, along with lower expression of MHC class I and interferon signaling, indicating an immune-cold RD microenvironment. Treatment of a basal-like and conventional chemotherapy-resistant PDX model, resembling basal-like RD, with sacituzumab govitecan or trastuzumab deruxtecan produced a marked antitumor response.

**CONCLUSION:**

RD biology differs from pretreatment tumors, with basal-like subtype RD following neoadjuvant chemotherapy being immune cold and associated with poor survival. Preclinical modeling suggests this high-risk group may benefit from adjuvant ADC therapy.

**TRIAL REGISTRATION:**

ClinicalTrials.gov NCT00861705.

**FUNDING:**

NIH NCI U10CA180821 (Alliance for Clinical Trials in Oncology), NCI U24CA176171 (Alliance for Clinical Trials in Oncology), NCI UG1CA233373 (Alliance for Clinical Trials in Oncology), NCI Breast SPORE program P50-CA058223; Susan G. Komen SAC-160074; Breast Cancer Research Foundation BCRF-23-127; NIH NCI R01-CA229409; UNC LCCC Triple Negative Breast Cancer Center.

## Abstract 2

Profiling post-chemotherapy residual disease revealed distinct biomarkers, identifying a high-risk, immune-cold, Basal-like group that showed responsiveness to antibody-drug conjugates in preclinical models.

## Graphical abstract

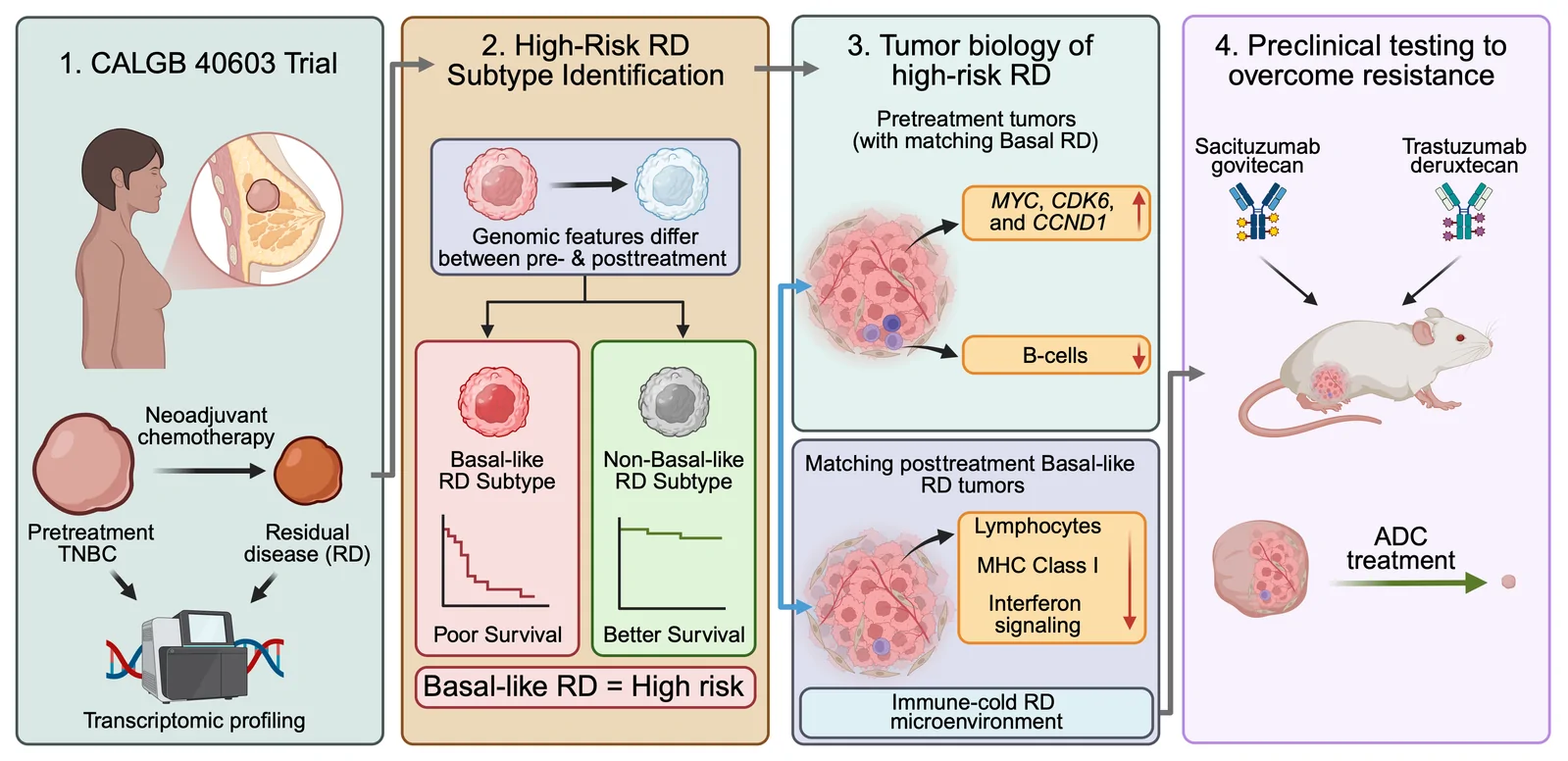

## Introduction

Patients with early-stage triple-negative breast cancer (TNBC) are commonly treated with neoadjuvant therapy, which, until recently, was exclusively chemotherapy based. It is well known that patients with TNBC who have residual disease (RD) following neoadjuvant therapy have a significantly worse prognosis compared with those with a pathological complete response (pCR) (1, 2). Although the addition of the anti–programmed cell death 1 (anti–PD-1) immune checkpoint inhibitor (ICI) pembrolizumab to neoadjuvant chemotherapy (NACT) has improved pCR rates and survival, approximately 35% of patients still exhibit RD at the time of surgery (3–5). These patients with RD are typically facing a worse prognosis due to therapy resistance and higher rates of early recurrence (6). Current efforts to improve the outcomes in this high-risk group of patients with RD include the use of adjuvant pembrolizumab and additional agents, such as capecitabine (5, 7). Emerging clinical strategies are increasingly focused on treatment escalation in patients with RD, with ongoing randomized trials evaluating trophoblast cell surface antigen 2-directed antibody-drug conjugate–based (ADC-based) regimens specifically in the RD TNBC setting, including sacituzumab govitecan plus pembrolizumab (OptimICE-RD/AFT-65/ASCENT-05, NCT05633654) and datopotamab deruxtecan with or without durvalumab (TROPION-Breast03, NCT05629585). Given that ADCs exert cytotoxic activity possibly independent of immune function, they represent a particularly attractive escalation strategy for patients with immune-cold RD, where the benefit of ICI monotherapy may be limited. The persistently poor prognosis of patients with TNBC RD underscores the urgent need to better characterize the molecular features of RD specimens. Despite the clinical and prognostic significance of RD, the biology of RD remains poorly defined, limiting our ability to guide and develop more effective post-NACT treatment strategies.

TNBC is the most aggressive clinical breast cancer subtype, with limited treatment options, largely because the tumors lack key therapeutic targets commonly present in other breast cancer subtypes — namely, estrogen receptor (ER) expression, progesterone receptor (PR) expression, and human epidermal growth factor receptor 2 amplification (2). In the absence of these targets, the current standard of care for early-stage TNBC relies on NACT combined with ICI to achieve a pCR prior to surgery. However, TNBC is a molecularly complex disease with multiple molecular tumor subtypes and microenvironmental differences, making a one-size-fits-all treatment approach challenging (2, 8). Gene expression profiling reveals substantial biological heterogeneity, with most TNBC tumors classified as basal-like by the PAM50 intrinsic subtype classifier (~75%) and the remaining tumors falling into the luminal A, luminal B, HER2-enriched, or normal-like molecular subtypes (9). TNBCs also vary widely in their immune landscape, often displaying high levels of tumor-infiltrating lymphocytes (TILs), programmed cell death ligand 1 (PD-L1) expression, and specific immune gene signatures (10–13). Particularly, gene expression signatures of adaptive immunity have shown prognostic value for early-stage TNBC beyond the standard clinical measures, such as tumor size, nodal involvement, and post-neoadjuvant pCR status (14).

Predictive and prognostic biomarkers in early-stage TNBC are typically derived from pretreatment tumor samples (15–18). However, tumor biology is dynamic, and treatment-induced tumor and immune changes can alter a tumor’s molecular phenotype (19–21), potentially influencing response to subsequent therapies and long-term outcomes. As a result, biomarkers identified before treatment may not fully capture the evolving biology of RD, necessitating the exploration of RD biology and associated posttreatment biomarkers to possibly refine clinical decision-making. Currently, the best prognostic tool in the posttreatment setting is the stratification of patients into residual cancer burden (RCB) groups, which is a measure of the extent of RD. Patients who achieve a pCR (RCB-0) or have minimal RD (RCB-I) have substantially better survival outcomes than patients with moderate (RCB-II) or extensive (RCB-III) RD following treatment (14, 22). Despite RCB’s prognostic value, it is currently not the standard of care, and it has been unclear if biological features of the RD can provide additional prognostic power.

The goal of this study was to characterize the biology of RD in the breast following NACT using RNA sequencing (RNA-seq) and to utilize the resulting molecular data to identify features associated with survival outcomes. To accomplish this, we analyzed the pretreatment and matching posttreatment gene expression profiles of TNBCs that were collected as part of the CALGB 40603 randomized phase II clinical trial (NCT00861705) (Cancer and Leukemia Group B, CALGB, is now part of the Alliance for Clinical Trials in Oncology, Alliance) (14, 23). CALGB 40603 evaluated the effects of adding carboplatin or bevacizumab to a NACT regimen of weekly paclitaxel, followed by dose-dense doxorubicin and cyclophosphamide, in patients with stage II/III TNBC. Using exclusively pretreatment biopsies of CALGB 40603, we previously showed that genomic and transcriptomic markers of immune activation were independently associated with increased pCR rates and improved survival (14, 24). Here, we specifically focused on paired pre- and posttreatment samples from patients with RD following NACT and tested the potential utility of antibody-drug conjugates (ADCs) in this context using patient-derived xenograft (PDX) models.

## Results

### Patient and tumor characteristics.

**Figure 1:**
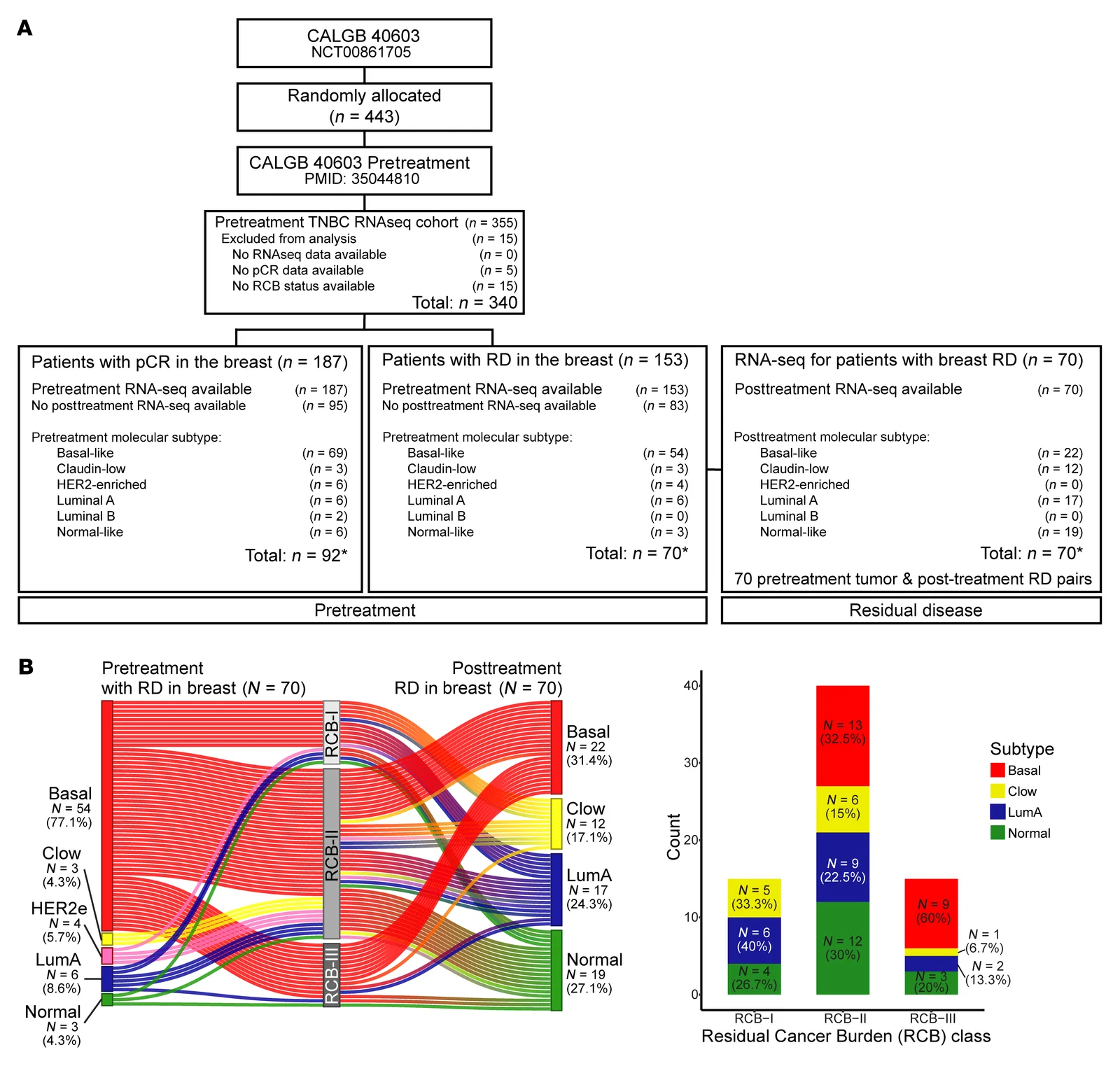
CONSORT diagram and molecular subtype characterization of RD. (**A**) CONSORT diagram for the available RNA-seq pretreatment and posttreatment samples for CALGB 40603. (**B**) Sankey diagram depicting molecular breast cancer subtypes in 70 patients with RD before and after treatment; residual cancer burden (RCB) class is shown as an intermediate node. (**C**) Stacked bar plot showing the distribution of posttreatment PAM50 molecular subtypes stratified by RCB class. Subtypes are color-coded as follows: basal = basal-like (red), Clow = Claudin-low (yellow), HER2e = HER2-enriched (pink), LumA = luminal A (blue), normal = normal-like (green).

We were interested in exploring the biology and possible prognostic significance of RD in patients with TNBC after NACT and thus performed gene expression profiling of pretreatment and posttreatment samples from CALGB 40603. This trial enrolled 454 patients, 443 (97.6%) of whom started treatment, and 426/443 (96.2%) were evaluable for the primary endpoint of pCR (Figure 1A). We obtained the gene expression profiles of pretreatment core biopsies from 355/426 (83.3%) patients, which have been previously analyzed and published (14, 24). Gene expression profiles from matching posttreatment breast specimens were procured from 166/355 (46.8%) patients. To perform analyses guided by clinical features, we excluded patients with missing pCR or RCB status from these matched pretreatment and posttreatment specimens, resulting in 340 patients with pretreatment tumors. Out of the 340 patients, 187 had a pCR in the breast following treatment (55.0%), and 153/340 (45.0%) had RD. Combined, 162/340 (47.6%) specimens had matching posttreatment specimens. Out of these 162 patients, 92 (56.8%) achieved a pCR in the breast and were not included in further analysis of posttreatment samples. The remaining 70 patients (43.2%) were reported to have RD in the breast, yielding 70 matched pairs of pretreatment and posttreatment RD specimens. No significant differences in clinicopathological characteristics, event-free survival (EFS), or overall survival (OS) were detected between all 340 pretreatment patients and the 162 pretreatment patients with matching pre- and posttreatment samples (Supplemental Figure 1, A–C; supplemental material available online with this article; https://doi.org/10.1172/JCI205962DS1). Subsequent analyses on pretreatment samples were therefore conducted on the 162 pretreatment samples with matching posttreatment samples, unless otherwise noted.

PAM50 subtypes were determined for all pretreatment samples and for the 70 posttreatment RD specimens. No pretreatment or RD subtype distribution differences by treatment arm were observed (Fisher’s exact test with Monte Carlo simulation, *P* = 0.91, and *P* = 0.4, respectively). Focusing on the 70 patients with RD, we identified a significant difference in molecular subtype frequencies between pretreatment and posttreatment samples (χ^2^ *P* < 0.0001). With 77.1% of pretreatment tumors, the basal-like subtype was the most frequent, followed by luminal A (8.6%), HER2-enriched (5.7%), and Claudin-low and normal-like (both 4.3%). While the basal-like subtype remained the most frequent (31.4%) in posttreatment RD, the frequency dropped substantially with a concomitant increase of normal-like (27.1%), luminal A (24.3%), and Claudin-low (17.1%) (Figure 1B). A global Fisher’s exact test indicated no association between treatment and subtype switching patterns (*P* = 0.45). Stratified analyses by pretreatment subtype also showed no significant differences in switching patterns (all adjusted *P* = 1.00; basal-like: *P* = 0.66; Claudin-low: *P* = 1.00; HER2-enriched: *P* = 1.00; luminal A: *P* = 0.50; normal-like not testable due to insufficient variation). Overall, no statistically significant treatment-specific differences in subtype switching were detected, though power is limited by sparse counts. Of the 54 basal-like pretreatment tumors with matching RD, 21 (38.9%) remained basal-like, while the others switched subtype into normal-like (25.9%), luminal A (20.4%), and Claudin-low (14.8%). Notably, 1 Claudin-low pretreatment tumor switched to basal-like in RD, resulting in 22 total patients with basal-like RD. The pretreatment subtype distribution between the tumors of patients with RD (Figure 1B) and the tumors of patients with a pCR in the breast (Supplemental Figure 2) showed no difference (χ^2^ *P* = 0.808). We also evaluated the 70 paired tumors in Figure 1B using the Lehmann et al. TNBCtype-6 and TNBCtype-4 subtyping methods (25, 26) (Supplemental Figure 3, A and B), both of which showed significant differences in subtype frequency between pre- and posttreatment samples (Fisher’s exact test with Monte Carlo simulation, *P* < 0.0001). For TNBCtype-4, however, this difference was largely driven by unevaluable RD samples, and their exclusion resulted in a nonsignificant subtype distribution (*P* = 0.21).

RD specimens in CALGB 40603 were further classified according to the RCB index, which is a validated prognostic feature in TNBC (27, 28). For patients with minimal RCB (RCB-I; *N* = 15), no specimens were identified as basal-like (Figure 1C). Instead, RCB-I specimens were predominantly luminal A (40%), Claudin-low (33.3%), or normal-like (26.7%). This observation does not rule out the possibility of microscopic basal-like RD in RCB-I specimens, as molecular subtypes are determined using PAM50 on gene expression data from bulk tissue, and detecting microscopic basal-like disease may fall below the bulk assay’s detection limit. The same limitation likely applies to other PAM50 subtype calls derived from low-tumor fraction RCB-I samples, meaning that PAM50 subtype assignments in this RCB class may not accurately reflect the underlying tumor subtype that may be identified using single-cell methods. Patients with moderate RD (RCB-II; *N* = 40) exhibited mostly basal-like RD (32.5%), followed by normal-like (30%), luminal A (22.5%), and Claudin-low (15%). Out of all patients with extensive RD (RCB-III; *N* = 15), 60% had basal-like RD, with the remaining 40% having normal-like (20%), luminal A (13.3%), and Claudin-low (6.7%) RD.

### Proliferation and PAM50 molecular subtypes are prognostic of survival in residual disease.

**Figure 2:**
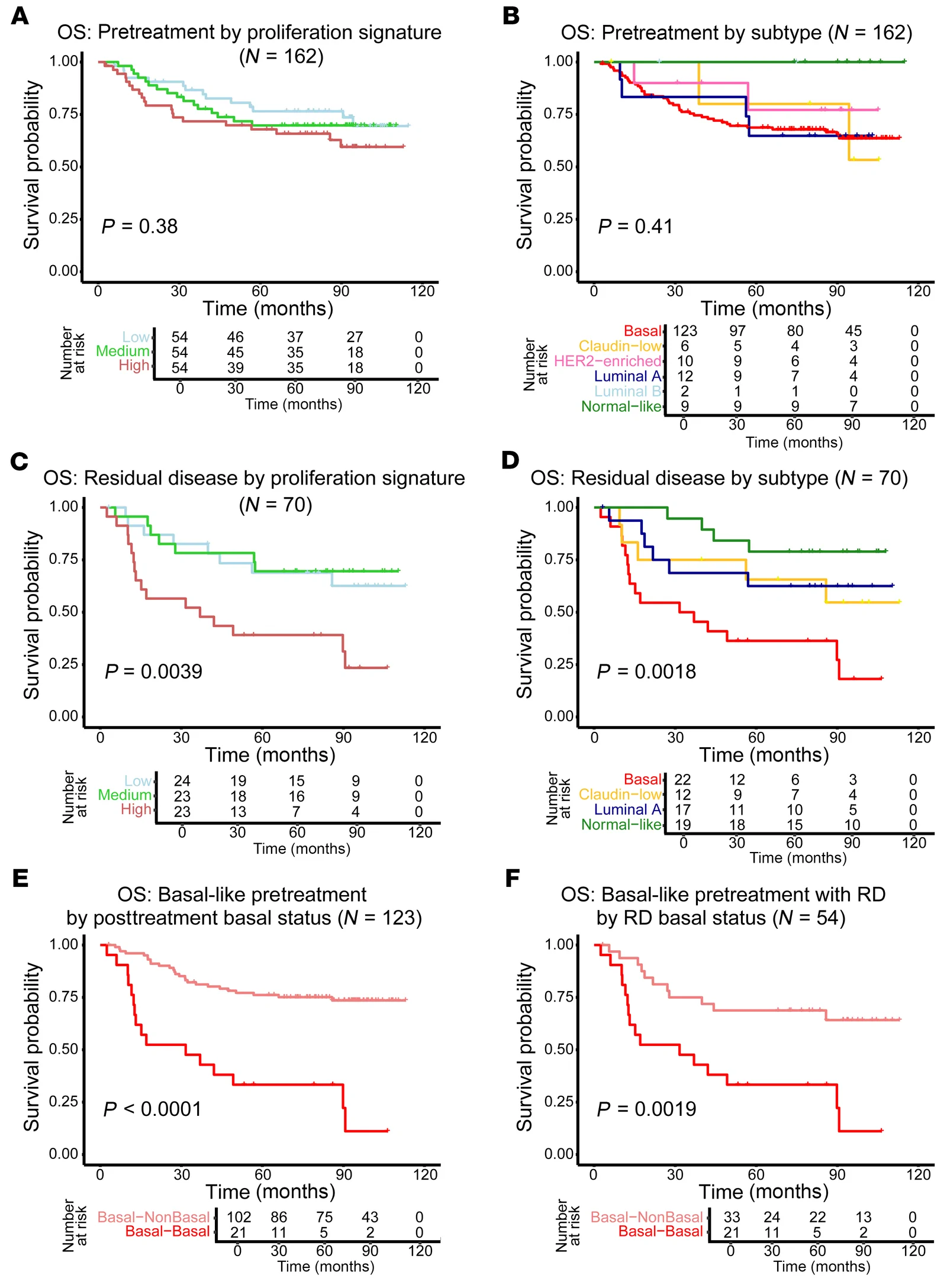
Kaplan-Meier curves of OS stratified by a proliferation gene expression signature and molecular breast cancer subtype. OS was analyzed in pretreatment tumors with matched posttreatment samples (*N* = 162) using tertiles of a proliferation signature (GSEA_GP1_Proliferation_DNA_repair.*r*=0.972_REACTOME_CELL_CYCLE_MITOTIC) (**A**) and molecular breast cancer subtype (**B**). Among tumors with RD (*N* = 70), OS was assessed by proliferation tertiles (**C**) and by molecular subtype (**D**). OS in patients with pretreatment basal-like tumors (*N* = 123) was stratified by posttreatment subtype — non–basal-like (*N* = 102, basal-nonbasal) versus basal-like (*N* = 21, basal-basal) (**E**) — and further restricted to pretreatment basal-like tumors with RD, comparing those that remained basal-like (*N* = 21, basal-basal) versus those that did not stay basal-like (*N* = 33, basal-nonbasal) (**F**). All *P*s were determined using the log-rank test. Five-year survival estimates, number of events, hazard ratios, and associated *P*s are presented in Supporting Data Values.

Messenger RNA-seq (mRNA-seq) gene expression analysis was performed on all RD specimens, after which we tested genomic features (*N* = 957) as potential prognostic biomarkers. We first investigated the prognostic utility of a proliferation signature (29) and of PAM50 intrinsic subtypes in pretreatment samples (*N* = 162) and matching posttreatment RD samples (*N* = 70). No OS or EFS differences by proliferation or intrinsic subtype were detected in pretreatment samples (Figure 2, A and B, and Supplemental Figure 4, A and B), which is consistent when testing all pretreatment tumors (*N* = 340) (Supplemental Figure 4, C–F). Similarly, we did not observe any prognostic value using the pretreatment TNBCtype-6 subtypes (log-rank test; OS *P* = 0.49, EFS *P* = 0.58) or the TNBCtype-4 subtypes (log-rank test; OS *P* = 0.78, EFS *P* = 0.7). Conversely, the molecular proliferation signature showed significant prognostic utility in RD samples (OS log-rank *P* = 0.0039) (Figure 2C), with highly proliferative samples having the poorest outcome (39.1% 5-year OS) compared with lowly or moderately proliferating samples (68.8% and 69.6% 5-year OS, respectively). We detected a significant OS difference between low-proliferation RD and high-proliferation RD (HR = 2.9; 95% CI: 1.24 to 6.81; *P* = 0.014) but no significant difference between low- and moderate-proliferation RD; this survival pattern by proliferation status was also observed for EFS among patients with RD (Supplemental Figure 4G).

Similarly, the OS of patients with RD was significantly different when stratified by RD intrinsic PAM50 subtypes (log-rank *P* = 0.0018) (Figure 2D), with patients with basal-like RD having the poorest outcome (36.4% 5-year OS). The 5-year OS for patients with Claudin-low RD was 65.6%, 62.5% for patients with luminal A RD, and 78.9% for patients with normal-like RD. Compared with normal-like RD, the OS of basal-like RD was significantly different (HR = 6.04; 95% CI: 2.00 to 18.19; *P* = 0.0014) but not for luminal A or Claudin-low RD; EFS survival patterns were consistent with those observed for OS in RD (Supplemental Figure 4H). Furthermore, we identified a significant association between the molecular proliferation signature and intrinsic molecular subtype (χ^2^ *P* < 0.0001), with 87.5% of all basal-like RD tumors falling into the high-proliferation group (Supplemental Figure 5). Among patients with basal-like pretreatment tumors (*N* = 123), those with basal-like RD (labeled basal-basal, *N* = 21) had a significantly reduced OS (log-rank *P* < 0.0001) (Figure 2E). The 5-year OS for those patients was 33.3% compared with 75.1% of patients whose pretreatment tumors were basal-like but nonbasal-like posttreatment (basal-nonbasal, *N* = 102). A significant increase in the HR for OS (HR = 4.86; 95% CI: 2.59 to 9.11; *P* < 0.0001) was observed in basal-basal patients compared with basal-nonbasal patients, indicating a higher risk of mortality is associated with patients who have basal-like RD posttreatment. This association remained significant in the subset of patients with basal-like pretreatment tumors that had RD in the breast posttreatment (*N* = 54), with a HR of 2.71 (95% CI: 1.57 to 7.43; *P* = 0.0019) (Figure 2F). Last, to evaluate whether incorporating RD basal-like status improved prognostic performance beyond RCB alone, we compared 2 Cox proportional hazard models: one including RCB alone and one including both RCB and RD basal-like status (i.e., basal-like or nonbasal-like). The model including basal-RD status demonstrated a significantly better fit for OS (likelihood ratio test, χ^2^ = 8.95, *df* = 1, *P* = 0.0028; degrees of freedom [*df*]) and EFS (likelihood ratio test, χ^2^ = 6.33, *df* = 1, *P* = 0.0119), indicating that basal-RD status provides additional prognostic information independent of RCB. We did not observe any significant associations between survival outcomes and the posttreatment TNBCtype-6 subtypes (log-rank test: OS *P* = 0.39, EFS *P* = 0.69) or the posttreatment TNBCtype-4 subtypes (log-rank test: OS *P* = 0.44, EFS *P* = 0.91). Consequently, subsequent analyses focused on the PAM50 subtypes.

### Molecular characterization of pretreatment tumors from patients with basal-RD.

**Figure 3:**
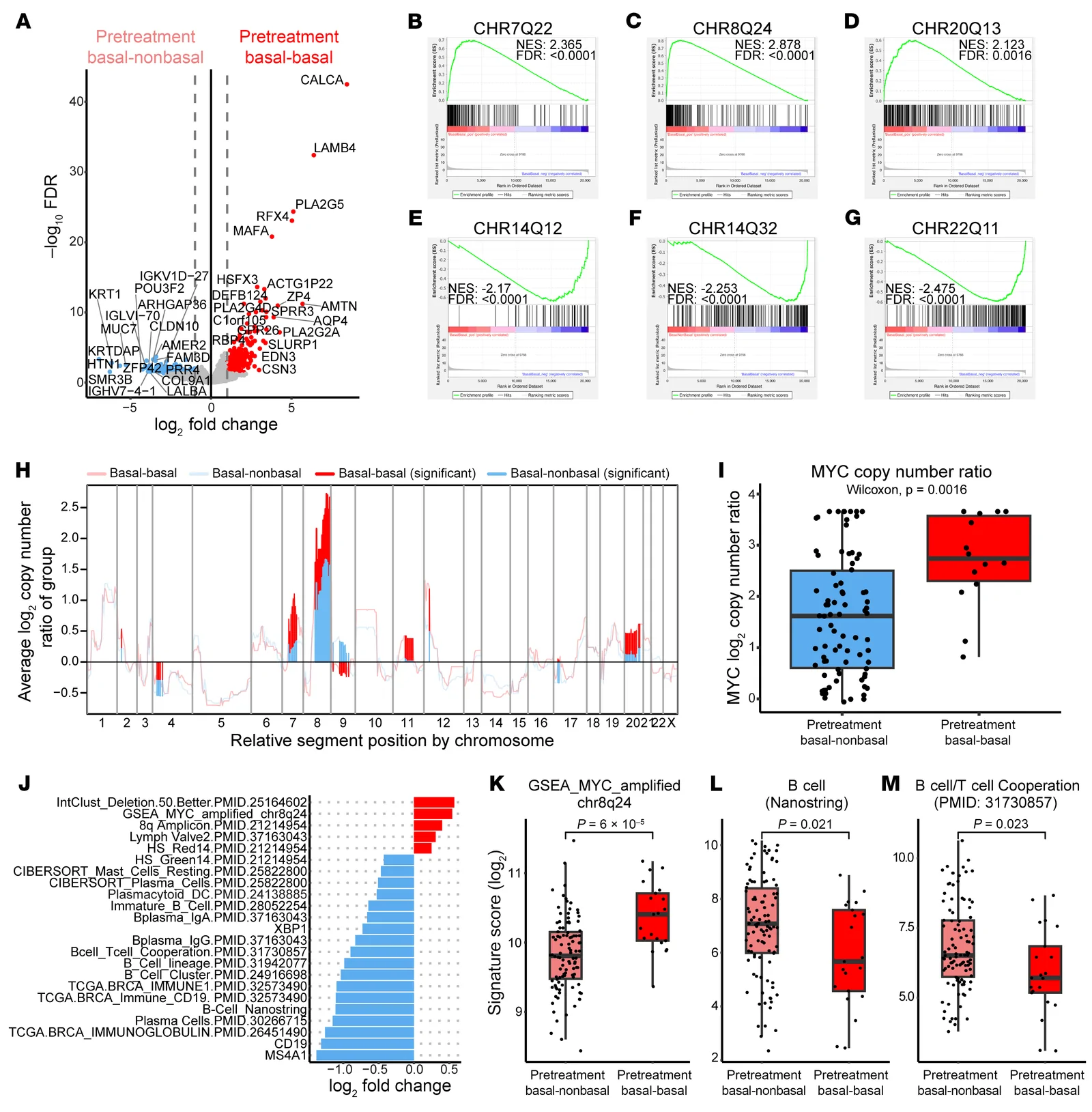
Comparative molecular profiling between basal-nonbasal and basal-basal pretreatment tumors. (**A**) Volcano plot illustrating differential gene expression between basal-nonbasal (*N* = 102) and basal-basal (*N* = 21) samples; genes significantly upregulated in basal-basal (FDR < 0.05, log_2_FC > 1) are highlighted in red, while those downregulated (FDR < 0.05, log_2_FC < –1) are shown in blue. (**B**–**G**) Gene set enrichment analysis (GSEA) plots displaying chromosomal segments with significant enrichment in basal-basal: (**B**–**D**) positively enriched regions (Chr7q22, Chr8q24, Chr20q13) and (**E**–**G**) negatively enriched regions (Chr14q12, Chr14q32, Chr22q11). (**H**) Bar plot representing the average log_2_ copy number ratio across samples; bold red bars indicate regions significantly (*P* < 0.05) amplified/deleted in basal-basal, whereas bold blue bars denote regions significantly amplified/deleted in basal-nonbasal. (**I**) Box plot of log_2_-transformed *MYC* copy number ratios comparing basal-nonbasal (blue) and basal-basal (red). (**J**) Bar plot of gene signatures differentially expressed between basal-nonbasal and basal-basal (*q* value < 0.05); signatures upregulated in basal-basal are shown in red, and those downregulated are in blue. (**K**–**M**) Box plots displaying log_2_ gene signature expression of MYC-amplified region Chr8q24 (**K**), B cell (**L**), and B cell/T cell cooperation (**M**) signatures for pretreatment basal-nonbasal (light red) and basal-basal (red) samples. For all box plots, individual data points are shown, and *P* values were calculated using the Wilcoxon rank-sum test.

Given the poor outcomes of patients with basal-like RD compared with patients with RD of other molecular subtypes, we sought to investigate how basal-like pretreatment tumors from patients with basal-like RD (basal-basal) compared with basal-like pretreatment tumors that were nonbasal-like in RD specimens after NACT (basal-nonbasal). Comparing the 21 basal-basal and 102 basal-nonbasal pretreatment tumors, using differential gene expression analysis, we identified 527 genes that were significantly higher and 200 genes that were significantly lower in basal-basal pretreatment specimens (Figure 3A; Supporting Data Values). Gene set enrichment analysis (GSEA) revealed a positive enrichment of genes in chromosomal regions in basal-basal tumors, including Chr7q22, Chr8q24, and Chr20q13, with the strongest enrichment for Chr8q24 (normalized enrichment score [NES] = 2.878; FDR < 0.0001) (Figure 3, B–D; Supporting Data Values). Amplifications on Chr8q24 and *MYC*, which is located on Chr8q24, have been previously associated with breast cancer and chemotherapy resistance (30). Among the negatively enriched chromosomal regions were Chr14q12, Chr14q32, and Chr22q11 (Figure 3, E–G). Using predetermined chromosomal segments (31), we identified that pretreatment basal-basal tumors (*N* = 14) also show higher amplification of segments that overlap with Chr7q22, Chr8q24, and Chr20q13 on the DNA level compared with pretreatment basal-nonbasal tumors (*N* = 82) (Figure 3H; Supporting Data Values). *MYC*, a prominent oncogene located on Chr8q24, also had significantly higher DNA amplification on the single-gene level (Figure 3I). Additional recurrent single-gene amplifications included *CDK6* (Chr7q21.3) and *CCND1* (Chr11q13.2) (Supporting Data Values). The gene segments on chromosomes 14 and 22 that were negatively enriched by GSEA were not significantly more deleted in basal-basal tumors on the DNA level. Notably, the leading-edge genes contributing to the Chr14 and Chr22 enrichments predominantly included immunoglobulin heavy and light chain genes. A notable single-gene loss in basal-basal samples is *CDKN2A/B* (Chr9p21.3). Given their common but antagonistic roles in Rb and cell cycle regulation, we evaluated whether there are any amplification/deletion patterns (i.e., mutual exclusivity) among *CDK6*, *CCND1*, and *CDKN2A/B* (Supplemental Figure 6) but found no statistical evidence for such patterns in this cohort (permutation test, *P* = 0.28).

To further investigate the differences between basal-basal and basal-nonbasal tumors, we compared them using previously published and publicly available gene expression signatures (*N* = 957) that comprehensively cover biological features of breast cancer (29, 32). Signatures with higher expression in basal-basal samples were associated with chromosomal amplifications of chromosome 8q (Figure 3J; Supporting Data Values). Specifically, the chromosomal region that carries the *MYC* gene, 8q24, was significantly upregulated in basal-basal tumors (Figure 3K), which is consistent with the GSEA and DNA-based chromosomal amplification findings. On the gene expression level, the *MYC* gene was not significantly higher but showed a trend toward increased expression (*P* = 0.062) (Supplemental Figure 7A). The most prominent lower signatures in basal-basal were immune features (i.e., B cells, T cells, and dendritic cells). Among those, the majority were B cell signatures. Specifically, the genes *XBP1*, *CD19*, and *MS4A1* (CD20), important in B cell biology, had lower expression in basal-basal tumors (Figure 3J). Additionally, immunoglobulin, B cell lineage, and the cooperation signature of B cells and T cells had lower expression in basal-basal tumors (Figure 3, J, L, and M). We observed no significant differences in CD8^+^ T cell signatures or in the expression of *CD274* (PD-L1) between the 2 groups (Supplemental Figure 7, B and C). Overall, these findings are consistent with previous publications that suggest patients with low B cell levels in their breast tumors have reduced EFS and OS (14, 33).

Next, we narrowed our focus to the 54 basal-like pretreatment tumors from patients who had RD following treatment (Figure 1B) to further investigate the differences in molecular tumor characteristics among patients with an adverse prognosis. We showed in Figure 2F that among those patients, basal-like RD remains a predictor of worse survival. We therefore performed a differential gene expression analysis followed by GSEA between the 33 (61%) basal-nonbasal and 21 (39%) basal-basal pretreatment tumors. We identified that the chromosomal amplification and deletion patterns remained consistent (Supplemental Figure 8, A–H; Supporting Data Values), including higher *MYC* copy number ratios in pretreatment basal-basal tumors (Supplemental Figure 8I). When comparing the 33 basal-nonbasal and 21 basal-basal pretreatment tumors using gene signatures, we did not identify any significantly higher (FDR < 0.05) signatures in the basal-basal cohort (Supplemental Figure 8J; Supporting Data Values). However, 90 signatures were significantly lower in the 21 basal-basal tumors. Several of these signatures were associated with luminal breast cancer characteristics, including *FOXA1*, *PGR*, and ER metabolite–associated gene expression. This finding was supported by comparing the correlation to the PAM50 centroid for the 2 groups, showing a significantly higher correlation to the luminal A centroid in basal-nonbasal while the correlation to other subtypes was not different (Supplemental Figure 9, A–E). However, the correlation to the luminal A centroid overall remained negatively correlated, and the strongest correlation remained with the basal centroid, suggesting that pretreatment basal-nonbasal tumors are strongly basal-like with a slightly higher expression of luminal features. While there were no significantly upregulated gene signatures by FDR in the basal-basal group in this comparison in this comparison, we tested the mRNA expression of *MYC* individually, which still showed a trend of higher expression in pretreatment basal-basal tumors (*P* = 0.1), and the gene signature for Chr8q24 remained significantly higher (*P* = 0.0033) (Supplemental Figure 8, K and L). The most prominent features that were lower in the pretreatment basal-basal tumors were luminal breast cancer and immune features (i.e., B cells, mast cells, and dendritic cells) (Supplemental Figure 8, J and M). Among those signatures, plasmacytoid dendritic cells (pDCs) were prominently lower in basal-basal tumors (Supplemental Figure 8N), and prior studies suggest that patients with TNBC whose tumors have higher levels of pDCs have a better outcome (34). Basal-basal tumors were also trending to have lower expression of the B cell/T cell cooperation signature (*P* = 0.081) and no significant differences in *CD274* (PD-L1) gene expression (Supplemental Figure 8, O and P), which is consistent with the comparison that includes patients with a pCR in the breast.

### Molecular characterization of treatment-resistant basal-like RD.

**Figure 4:**
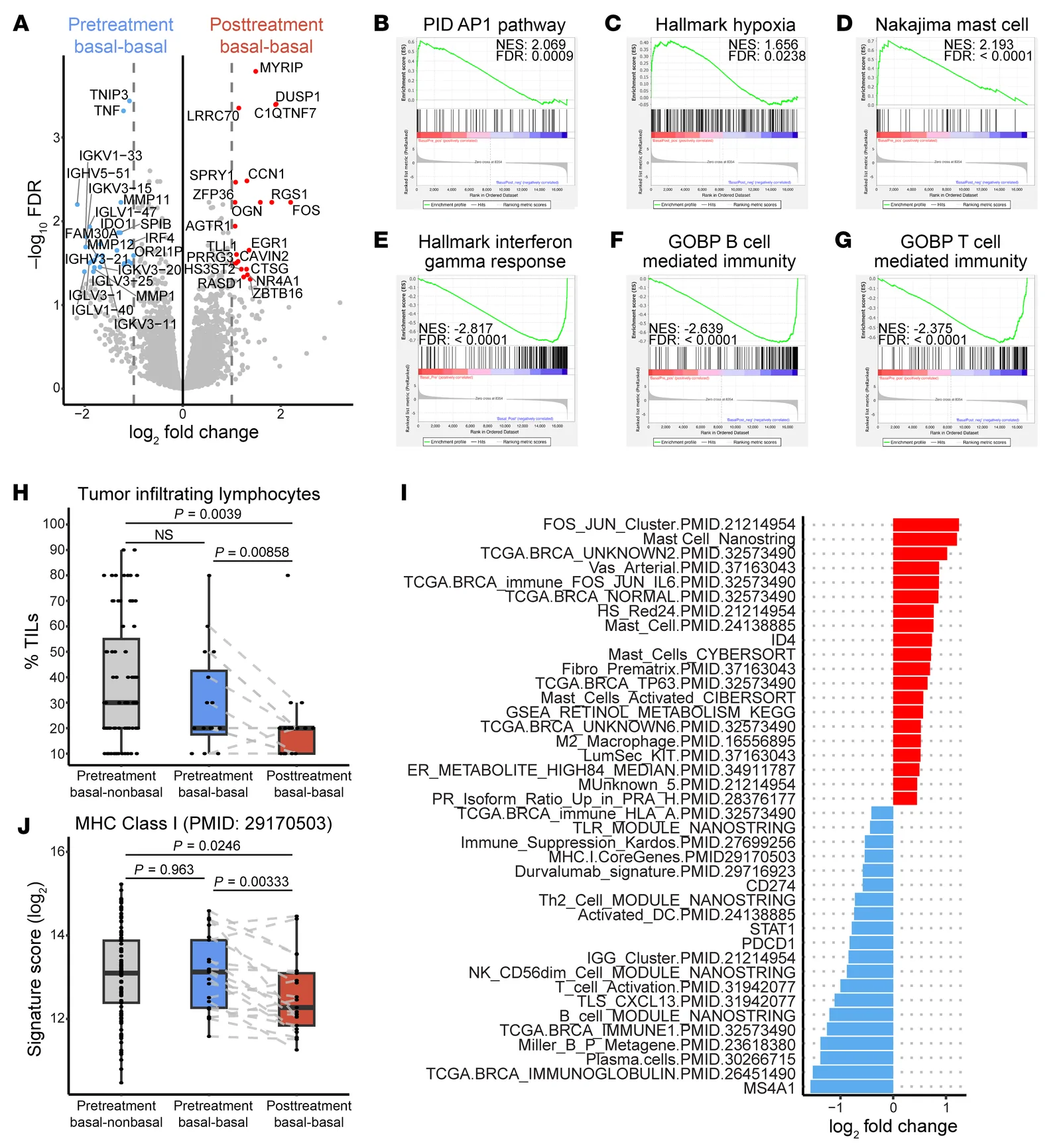
Transcriptomic and immune changes in matched basal-like tumors pre- and posttreatment. This figure compares 21 matched tumor samples classified as basal-like both pre- and posttreatment. (**A**) Volcano plot of differentially expressed genes, with genes expressed higher in posttreatment (FDR < 0.05, log_2_FC ≥ 1) in red and lower expressed genes (FDR < 0.05, log_2_FC ≤ –1) in blue. (**B**–**G**) GSEA plots showing representative pathways enriched posttreatment (**B**–**D**: AP-1 signaling, hypoxia, mast cells) and negatively enriched (**E**–**G**: interferon-gamma response, B cell–mediated immunity, T cell–mediated immunity). (**H**) Histopathologic TIL levels across pretreatment basal-nonbasal (pretreatment basal-like tumors that were not basal-like posttreatment, gray), pretreatment basal-basal (pretreatment basal-like tumors that remained basal-like posttreatment, blue), and matching posttreatment basal-like RD samples (red) (*N* = 12 matched pairs). (**I**) Bar plot of top significantly altered gene signatures (*q* < 0.05), with red indicating higher expression and blue lower expression in posttreatment basal-like RD. (**J**) Box plot of log_2_ gene signature expression for the MHC class I signature in pretreatment basal-nonbasal, pretreatment basal-basal, and matching posttreatment basal-basal samples. For box plots individual data points are shown, and *P* values were determined using Wilcoxon rank-sum test for pretreatment basal-nonbasal versus pretreatment basal-basal and pretreatment basal-nonbasal versus posttreatment basal-basal groups and paired *t* tests for pretreatment basal-basal versus their matched posttreatment samples.

We next sought to compare the basal-basal pretreatment tumors with their matching basal-like RD specimens (*N* = 21 pairs). In posttreatment basal-like RD, 48 genes had significantly higher expression, and 69 genes were significantly lower expressed when compared with their matching pretreatment samples (Figure 4A; Supporting Data Values). Among the genes with lower expression were predominantly immunoglobulin genes and genes that are associated with immune response regulation, including *MMP1*, *TNF*, *TNIP*, *ZBP1*, *IDO1*, and *IRF1*. GSEA revealed that the higher expressed genes in posttreatment basal-like RD are associated with several features, including the AP-1 pathway, hypoxia, and mast cells (Figure 4, B–D; Supporting Data Values). Prominent features with lower expression in basal-like RD were interferon response, B cell–mediated immunity, and T cell–mediated immunity (Figure 4, E–G; Supporting Data Values). Consistent with these findings, histological assessment demonstrated a corresponding decrease in TILs in the basal-like RD samples (Figure 4H).

Using our panel of gene signatures, we identified a significant increase of FOS/JUN signaling, arterial vasculature, mast cells, EGF signaling, and M2 macrophages in basal-like RD (Figure 4I and Supplemental Figure 10, A–D; Supporting Data Values). Another elevated signature, TCGA.BRCA.1198_NORMAL (35), suggested that there is an increase in normal-like breast cancer features in basal-like RD. We further explored this by evaluating the PAM50 subtype score (correlation to the centroid) in pretreatment and paired posttreatment basal-like tumors. While the correlation to the basal-like and luminal B centroid were unchanged (Supplemental Figure 11, A and D), there were a significant decrease in the correlation to the HER2-enriched centroid (*P* = 0.0273) (Supplemental Figure 11B) and a significant increase in centroid correlation of both luminal A (*P* = 0.0162) and normal-like (*P* = 0.0269) (Supplemental Figure 11, C and E). *MYC* gene expression was not increased at a statistically significant level in basal-like RD compared with matching pretreatment samples (Supplemental Figure 10E). However, *MYC* was significantly higher expressed in basal-like RD in comparison with basal-like pretreatment tumors with nonbasal-like RD (*P* = 0.0123). The lack of DNA-seq for most of the basal-like RD samples prevented us from looking at chromosomal changes between pre- and posttreatment samples. Most downregulated genomic signatures in basal-like RD were associated with immune features, particularly adaptive immunity (Figure 4I). Both the MHC class I gene signature and the PD-L1 gene (*CD274*) showed significantly reduced expression after NACT in basal-like RD (Figure 4J and Supplemental Figure 10F), features that were not significantly different between basal-basal and basal-nonbasal tumors prior to treatment. A closer look at the expression of the individual genes that make up the MHC class I gene signature revealed that genes associated with antigen presentation (i.e., *HLA-A*, *HLA-B*, *HLA-C*, and *B2M*) were not significantly different, while genes associated with antigen processing (i.e., *TAP1*, *TAP2*, *PSMB8*, and *PSMB9*) were significantly down or strongly trending down in posttreatment basal-basal RD (Supplemental Figure 12). Notably, despite pretreatment basal-basal tumors already showing significantly lower levels of genomic B cell features in comparison with pretreatment basal-nonbasal tumors, we observed an even further decrease in B cell–associated signature expression and B cell/T cell cooperation signature expression in basal-like RD (Supplemental Figure 10, G and H). Additionally, T cell activation features that were not different between pretreatment basal-basal and basal-nonbasal were decreased in basal-like RD (Supplemental Figure 10I).

### Comparison of prognostic signatures between pretreatment and RD tumors.

**Figure 5:**
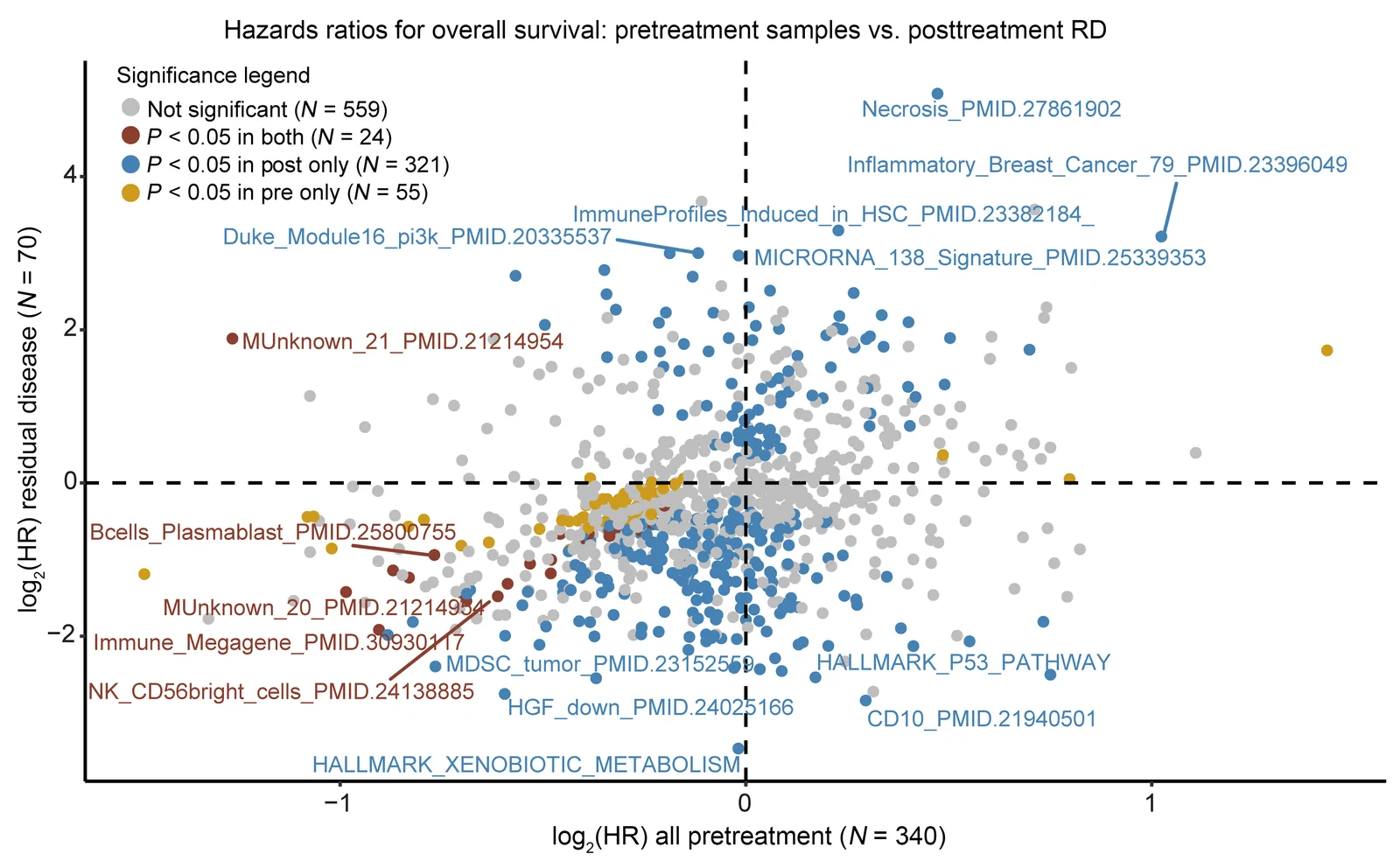
Comparison of Cox model HRs for OS in pretreatment samples and posttreatment RD using gene expression signatures (*N* = 959). HRs for all pretreatment tumors (*N* = 340) versus RD samples (*N* = 70). HRs are derived from Cox proportional hazard modeling using gene expression signature expression as continuous covariate. Pretreatment HRs are plotted on the *x* axis and RD HRs on the *y* axis. Colored points indicate gene signatures significantly associated with OS (*P* < 0.05): maroon denotes signatures significant in both pretreatment and RD samples, yellow indicates significance in pretreatment only, and blue indicates significance in RD only. Axes display log_2_-transformed HRs.

Using all pretreatment TNBC samples from CALGB 40603, we have previously shown that B cell– and T cell–associated gene expression signatures are predictive of EFS, with patients with higher B cell–associated expression having a better survival outcome (14). To identify if these features are predictive of survival in the posttreatment samples as well, we compared the survival outcome based on gene expression signatures using pretreatment TNBC (*N* = 340) and RD (*N* = 70) specimens separately, using univariate Cox proportional hazard models to identify features that may be shared or unique. Comparing the overlap between significant prognostic features (*P* < 0.05, unadjusted) for OS, we identified 24 gene expression signatures that are prognostic in pretreatment and in RD samples (Figure 5; Supporting Data Values). Of the 24 common signatures, 23 (96%) were associated with favorable clinical outcomes, and most of them represent immune-related features, including naive B cells, plasmablasts, and multiple T cell subset signatures. For EFS, the overlap of prognostic features was lower, with only 4 signatures prognostic in pretreatment samples and posttreatment RD (Supplemental Figure 13; Supporting Data Values). We did not identify a clear biological pattern for these overlapping features; however, 3 of the 4 features (MUnknown_20_ PMID.21214954, CD8_Tem_PMID.37163043, Bnaive_PMID.37163043) were shared with the overlapping OS features (Supplemental Figure 14A). We observed that gene expression signatures prognostic of OS in both pre- and posttreatment were more characteristic of early-stage B cell development (i.e., naive B cells and plasmablasts), while B cell features prognostic solely in pretreatment samples represented late-stage B cell characteristics (i.e., plasma cells, IgG-secreting). T cell signatures that were prognostic of OS in pre- and posttreatment were enriched for tissue-resident, memory, and helper T cell phenotypes, including CD8^+^ Trm and CD4^+^ Tfh, Th2, and naive subsets. In contrast, T cell signatures that were prognostic only in pretreatment samples were more strongly associated with cytotoxic effector CD8^+^ T cells, Th1 responses, and T cell activation.

Gene signatures that were solely prognostic in the posttreatment RD setting were substantially different from those predictive in the pretreatment setting. Specifically, signatures of mammary stem cell (MaSC) and stromal signatures were more frequent and predicted better OS and EFS in RD. Worse OS and EFS outcome in RD samples was predominantly associated with proliferation and MAPK signaling. These findings raised the possibility that the associations with survival may reflect underlying differences in RCB, which includes a direct measure of residual cancer cellularity in the breast. We therefore hypothesized that at least some features linked to improved outcomes, such as stromal and MaSC signatures, may indicate lower tumor content, while those associated with poorer prognosis, particularly proliferative signals, may reflect higher tumor cell content. To test this possibility, we examined the distribution of relevant signature scores across different RCB classes. MaSC signatures did not differ significantly between RCB-I and RCB-II samples but were significantly reduced in RCB-III tumors (Supplemental Figure 15A). Stromal signatures progressively and significantly declined with increasing RCB class (Supplemental Figure 15B). In contrast, proliferation signatures increased considerably in higher RCB classes (Supplemental Figure 15C). Indeed, after adjusting the Cox model for RCB class, 124 of the 346 gene signatures that were initially significant for OS in the RD setting lost statistical significance (Supplemental Figure 16; Supporting Data Values). Among the 124 signatures were 6 proliferation and 13 stromal signatures (Supplemental Figure 15D; Supporting Data Values). Notably, MaSC signatures remained largely significant as a predictor of better OS. We observed the same trend for EFS (Supplemental Figure 15E; Supporting Data Values). To explore which gene signatures may be prognostic across all RCB groups, we used a Cox model–based approach with RCB class as interaction term. Among the 12 shared prognostic signatures for OS (Supplemental Figure 15F; Supporting Data Values), 9 were directly linked to interferon signaling. In RCB-II and RCB-III, higher expression of these signatures was prognostic of better outcome, while in RCB-I they were prognostic of worse outcome (Supporting Data Values). Due to the limited sample sizes in the RCB-I (*N* = 15) and RCB-III (*N* = 15) groups, we did not further investigate which signatures were uniquely prognostic within the individual RCB subsets.

### ADC treatment of basal-like PDX tumors.

ADCs, such as sacituzumab govitecan (SACI), datopotamab deruxtecan (Dato-DXd), and trastuzumab deruxtecan (T-DXd), are currently being investigated in the neoadjuvant and adjuvant settings to further improve pCR rates, recurrence rates, and survival outcomes of patients with TNBC (e.g., NCT06112379, NCT05633654, NCT05629585, NCT04434040). To explore the potential of ADCs in this context, we tested SACI, T-DXd, and carboplatin plus paclitaxel (Carbo/Tax) in 2 basal-like TNBC PDX models, each with known clinical response to the therapies that the donor patients received.

**Figure 6:**
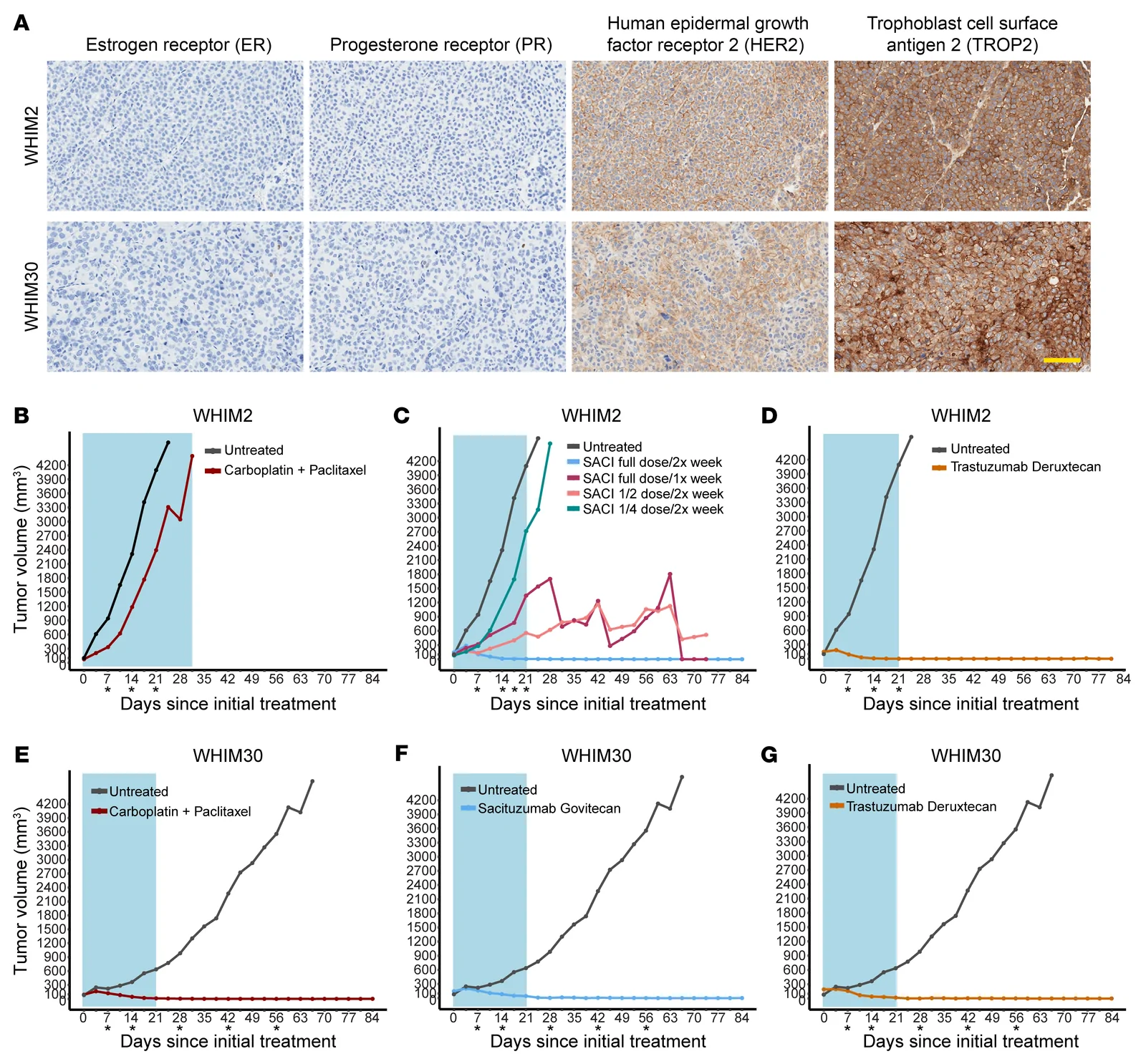
Immunohistochemistry and tumor growth curves for PDX models WHIM2 and WHIM30 treated with the ADCs SACI (Trodelvy) and T-DXd (Enhertu). (**A**) Immunohistochemistry staining of untreated WHIM2 and WHIM30 tumor sections with ER, PR, HER2, and TROP2. Representative images are shown at 40× original magnification, and scale bar indicates 100 μm. (**B**) WHIM2 untreated (gray, *N* = 23) and treated with carboplatin (50 mg/kg) and paclitaxel (9 mg/kg) once weekly (red, *N* = 8) until endpoint. (**C**) WHIM2 untreated (gray, *N* = 23), treated with SACI (blue, 10 mg/kg twice weekly for 3 weeks, *N* = 53), once weekly at standard dose (red, 10 mg/kg, *N* = 5), twice weekly at half dose (pink, 5 mg/kg, *N* = 5), or twice weekly at quarter dose (green, 2.5 mg/kg, *N* = 5), all until complete regression or tumor endpoint. (**D**) WHIM2 untreated (gray, *N* = 23) and treated with T-DXd (orange, 5 mg/kg twice weekly for 3 weeks, *N* = 18). (**E**) WHIM30 untreated (gray, *N* = 19) and treated with carboplatin and paclitaxel once weekly for 3 weeks (red, *N* = 20). (**F**) WHIM30 untreated (gray, *N* = 19) and treated with SACI (blue, 10 mg/kg twice weekly for 3 weeks, *N* = 29). (**G**) WHIM30 untreated (gray, *N* = 19) and treated with T-DXd (orange, 5 mg/kg twice weekly for 3 weeks, *N* = 17). **B**–**D** and **E**–**G** represent the same untreated control WHIM2 or WHIM30 mouse cohort, respectively. SACI, sacituzumab govitecan (Trodelvy); T-DXd, trastuzumab deruxtecan (Enhertu). Data points represent mean tumor volume, and the corresponding SEMs are reported for data points that were statistically tested. Stars indicate time points of statistical testing (Wilcoxon rank-sum test; Supporting Data Values).

To better understand the baseline characteristics of these PDX tumors, we performed RNA-seq on untreated tumors of the PDXs, WHIM2 and WHIM30 (36, 37), and integrated them into the CALGB 40603 dataset. Particularly, WHIM2, which was established from a pretreatment biopsy of a basal-like TNBC from a patient who ultimately had extensive RD following conventional NACT (36), displayed similar gene expression patterns to basal-like RD. WHIM2 showed elevated *MYC* (Supplemental Figure 17A) and low levels of tumor cell–associated *CD274* (Supplemental Figure 17B). WHIM30, on the other hand, showed relatively low levels of *MYC* gene expression and highly elevated levels of *CD274*. However, for each PDX model, gene expression of the SACI target *TACSTD2* (TROP2) (Supplemental Figure 17C) and the T-DXd target *ERBB2* (HER2) (Supplemental Figure 17D) was high and on par with what was seen in the various tumor subsets coming from CALGB 40603, with only minor decreases observed in WHIM30, which still exhibited high expression levels. Using immunohistochemistry on untreated WHIM2 and WHIM30 tumor sections, we confirmed that HER2 and TROP2 are expressed on the cell surface at the protein level (Figure 6A). ER and PR staining were absent in these tumors, confirming their TNBC status.

WHIM2 was derived from a TNBC inflammatory breast cancer of a 44-year-old woman prior to NACT (36). The patient received neoadjuvant dose-dense chemotherapy but had substantial RD at the time of mastectomy, indicating resistance to treatment. Consistent with this, Carbo/Tax treatment in WHIM2 PDX tumor–bearing mice resulted in a modest delay in tumor growth, but the tumors were ultimately resistant to the regimen (Figure 6B). We tested 4 SACI treatment schedules/doses on WHIM2 tumors, including a dose of 10 mg/kg administered twice a week, which best mimics the human dosing. At this dose all WHIM2 tumors completely regressed, and no recurrence was detected in any mice 8 weeks after treatment termination (Figure 6C). Mice that received half the standard dose (5 mg/kg) twice weekly, or the standard dose once a week, showed substantial heterogeneity in response and none achieved tumor control (Figure 6C). While T-DXd is currently not approved in the neoadjuvant setting for TNBCs, we wanted to explore its potential efficacy in WHIM2. All T-DXd–treated WHIM2 tumors completely regressed and did not recur within the 8-week period after the last administered dose (Figure 6D).

The second PDX, WHIM30, was derived from a *BRCA2*-mutant TNBC tumor of a 35-year-old woman prior to neoadjuvant treatment. The patient received neoadjuvant paclitaxel for 4 cycles, followed by 4 cycles of adriamycin/cytoxan, and achieved a pCR. This is mirrored by our treatment of WHIM30 PDX tumor–bearing mice with Carbo/Tax, where all mice showed complete tumor regression and long-term tumor-free survival (Figure 6E). In addition, the WHIM30 tumors that were treated with SACI (Figure 6F) or T-DXd (Figure 6G) also showed complete tumor regression, and no recurrence was observed within 8 weeks after the last dose.

## Discussion

RD after neoadjuvant therapy remains a major driver of poor outcomes in early-stage TNBC, despite the incorporation of ICIs into the standard of care (3–5). While the KEYNOTE-522 trial demonstrated that pembrolizumab can increase pCR rates and shift the overall distribution of RD toward lower RCB classes, patients with RCB-III disease may still not benefit from the addition of ICI (22). In this study we identified that patients with basal-like RD after non-ICI NACT have the worst survival, and here we characterize the biology of these specimens with the hope that these results can inform future clinical decision-making.

An important finding of our study is that patients with basal-like RD, all of which are RCB-II or RCB-III, have the worst survival outcomes, showing that molecular subtype in RD is prognostic of EFS and OS and may be able to serve as a biomarker in this setting. Importantly, this basal-like RD biomarker is independently prognostic of RCB status, suggesting the 2 together would be a powerful prognostic feature. Although the intrinsic molecular subtype is not currently assessed in the posttreatment setting, our data indicate that its incorporation could meaningfully enhance prognostic stratification. Pretreatment tumors from these high-risk patients exhibited elevated *MYC* amplification and *MYC* signature levels, and lower lymphocyte-associated gene expression, consistent with prior studies linking these features to poor clinical outcomes. Notably, it appears that these features are biologically connected, with studies showing that *MYC* can lead to an immunosuppressive microenvironment (30). We also identified recurrent copy number gains on chromosomes 7, 11, and 20, with the most prominent amplifications observed on chromosome 7. Specifically, *CDK6*, located on Chr7q21, was the most significantly amplified locus (*P* = 0.00130; Supporting Data Values), and its gene expression levels were elevated in basal-basal samples, suggesting a potential mechanism for cell cycle deregulation in these tumors. Elevated CDK6 protein levels have been previously observed in a subset of basal-like TNBCs that show poor response to chemotherapy (12). We additionally observed other amplifications and deletions (i.e., *CCND1* and *CDKN2A/B*, respectively) in basal-basal specimens that are associated with Rb signaling and cell cycle regulation, but there were no consistent gain/loss patterns associated between *CDK6*, *CCND1*, and *CDKN2A/B*, suggesting that their role in basal-like TNBC may be complex, and further studies will be needed to identify their potential role in chemotherapy treatment resistance.

Another objective of this study was to characterize changes between basal-like RD and matched pretreatment tumors. Basal-like RD exhibited higher expression of several pathways linked to tumor progression and therapeutic resistance, including hypoxia, arterial vasculature, and AP-1 (FOS/JUN) gene signature expression (38–40). These alterations are consistent with mechanisms described in other tumor types, where persistent hypoxia and aberrant vascular remodeling promote a dysfunctional microenvironment that impairs drug delivery and fosters aggressive tumor phenotypes (41). Additionally, basal-like RD demonstrated pronounced lower immune cell abundance, with significant reductions in lymphocyte-associated gene expression (i.e., B cell and T cell signatures) and reduced TIL levels. Furthermore, basal-like RD exhibits features of significant immunosuppression, characterized by substantially reduced antigen presentation–associated gene expression (i.e., MHC class I), inflammatory signaling (i.e., *TNF* and *INF*), and immune checkpoint genes (i.e., PD-L1). Interestingly, the reduced MHC class I signature expression was driven by lower levels in immunoproteasome genes (*PSMB8/9*) and peptide transport genes (*TAP1/2*) that shuttle peptides from the cytosol to the endoplasmic reticulum for loading onto MHC class I molecules, while genes associated with antigen presentation remained unchanged (*HLA-A/B/C* and *B2M*). Basal-like RD may therefore rely on impaired antigen processing rather than downregulated antigen presentation as a contributor of immune escape. Notably, this immune suppression occurred despite already relatively low baseline lymphocyte gene expression prior to treatment. These data align with the established concept that disruption of antigen presentation, TNF signaling, and IFN-γ cytokine signaling drives tumor immune evasion (42). Consequently, chemotherapy-induced suppression of antigen presentation and inflammatory signals in basal-like RD may further reinforce an immune-deprived phenotype by hindering effector lymphocyte activation and recruitment. Given these features, it remains uncertain whether these basal-like RD tumors would be sensitive to ICIs such as pembrolizumab (43). The marked immune suppression observed in RD, including impaired antigen presentation and reduced inflammatory signaling, may limit ICI efficacy by constraining T cell priming, activation, and infiltration.

These findings underscore the urgent need for therapeutic strategies capable of overcoming or bypassing immune exclusion, possibly by targeting immune-independent vulnerabilities. One such strategy may be the use of ADCs. In the metastatic TNBC setting, SACI has shown meaningful clinical benefit and is approved for use (44). Similarly, T-DXd is approved for a variety of breast cancer subsets, including unresectable or metastatic HER2-low (IHC 1+ or IHC 2+/ISH–) tumors (45). In this study, we show that WHIM2, a chemotherapy-refractory basal-like tumor, and WHIM30, a chemotherapy-sensitive basal-like *BRCA2*-mutant tumor, are both sensitive to SACI and T-DXd when grown in NSG mice. These mice are deficient in mature lymphocytes (i.e., B cells, T cells, and NK cells) and exhibit defects in cytokine signaling (46, 47), at least partially recapitulating the immune-suppressed microenvironment characteristic of basal-like RD. The efficacy of ADCs in these models suggests that their activity does not require an intact immune response.

The immune-suppressed microenvironment observed in basal-like RD in our study raises important questions about the potential benefit of adjuvant pembrolizumab monotherapy in this high-risk group. Several studies suggest that adjuvant pembrolizumab by itself has little to no impact on survival outcomes (48). With basal-like RD showing impaired antigen presentation, scarcity of immune effector cells, and dampened inflammatory signaling, pembrolizumab’s therapeutic efficacy may be limited. Taken together, these findings suggest that basal-like RD may be poorly suited for immune monotherapy but may be vulnerable to ADC monotherapy or a combination of the 2 therapy approaches. Several ongoing trials are exploring the combination of ADCs with ICIs in patients with RD after neoadjuvant treatment (NCT05633654, NCT05629585, NCT04434040).

One of the limitations of this study is that patients in this trial did not receive pembrolizumab, or another ICI, in combination with NACT, which is now the standard of care. This trial was conducted at a time when NACT only was the standard of care in the neoadjuvant setting for early-stage TNBC. Regardless, this study provides important insight into the molecular characteristics of tumors that persist following NACT alone, offering a critical foundation for understanding resistance biology and informing future therapeutic strategies, including those incorporating immunotherapy. Another limitation was the relatively low number of basal-like RD samples (*N* = 21), limiting our ability to assess prognostic molecular signatures in this high-risk group. Future studies with larger cohorts and inclusion of patients treated with NACT plus pembrolizumab will be critical to extend these findings. This is particularly important given the scarcity of publicly available paired RNA- and DNA-seq data from pre- and posttreatment TNBC samples, which precluded independent validation of our results, and simultaneously underscores the unique value of our dataset as a foundation for future studies.

In conclusion, patients with basal-like RD after NACT represent a profoundly high-risk TNBC subgroup characterized by chromosomal amplifications of *MYC* and cell cycle control genes and pronounced immune depletion. These features not only confer poor survival but also suggest limited benefit from ICI monotherapy and highlight alternative vulnerabilities, most notably to ADCs and potentially to cell cycle–targeted agents. Our findings thus provide a molecular rationale for prioritizing ADC-based strategies, alone or in combination with immunotherapy, to overcome the immune-suppressed microenvironment of basal-like residual tumors.

## Methods

### Sex as a biological variable.

Our study exclusively included female patients from the CALGB 40603 trial (NCT00861705), and only female mice and tumors derived from females were used in our PDX studies because breast cancer is a predominantly female disease.

### Trial design and patients.

Patient characteristics, trial design, and definitions of pCR, EFS, and OS were previously reported (14, 23). RCB was calculated for patients with RD per Symmans et al. (27). The study database was locked on April 2, 2020, with censoring based on the most recent follow-up.

### Tumor collection and sequencing.

Pretreatment core biopsies were collected from all patients for DNA-seq, RNA-seq, and histological assessment. Matched posttreatment tumor samples were also obtained for DNA-seq, RNA-seq, and histology from 166 patients after NACT. DNA-seq and RNA-seq followed protocols from Ciriello et al. (49), excluding samples that failed quality thresholds or showed hormone receptor expression greater than 1%, consistent with current TNBC definitions (50).

For RNA-seq, FASTQ files from pre- and posttreatment samples were processed by the UNC Lineberger Bioinformatics Core. Reads were aligned to the GRCh38/hg38 genome (GENCODE v36, RRID:SCR_014966) using STAR (v2.7.6a, RRID:SCR_004463), and transcript abundance was estimated with Salmon (v1.4.0). Quality control was conducted with Picard (v2.22.4, RRID:SCR_006525) and FastQC (v0.11.9, RRID:SCR_014583). Raw gene counts were normalized via upper quartile normalization and log_2_-transformed (count +1) for downstream analysis. For DNA-seq, data were processed as described by Felsheim et al. (24).

Untreated PDX tumors (~0.5–0.7 mm) and treated PDX tumors were collected as previously described (35) and sequenced/processed as described above.

### RNA expression analyses.

Differential gene expression analysis was performed using edgeR (v4.2.2, RRID:SCR_012802) (33) on raw gene counts, comparing pretreatment basal-basal tumors with either pretreatment basal-nonbasal tumors (unpaired) or matched posttreatment basal-like RD samples (paired). Genes with FDR < 0.05 were considered significant. GSEA was conducted using the Mac GSEA software (v4.3.3) on preranked gene lists, ranked by the sign of log fold-change × –log_10_(*P* value). Lists were analyzed against 5 reference gene sets (Hallmark, Positional, Curated, Ontology, and Cell Type), with significant enrichment defined as FDR *q* < 0.05.

Using normalized RNA expression data, scores were calculated for 959 published or public gene expression signatures covering pathways, cell types, disease states, and individual genes. A subset was previously described (51); full details are in Supporting Data Values. Signature scores were the median expression of genes in each signature. The samr R package (v3.0) was used to evaluate differences in signature score expression, with the “two-class unpaired” or “two-class paired” setting as appropriate. Significance was set at *q* < 0.05.

### Intrinsic subtype assignment.

The molecular breast cancer subtype of the pretreatment samples was determined using PAM50 as previously described (52). For the 166 posttreatment samples, subtypes were assigned by individually appending each sample to the pretreatment cohort prior to normalization, followed by classification using the standard PAM50 pipeline. This approach was selected to reduce potential biases introduced by lower tumor cellularity in a subset of posttreatment specimens. In addition to the PAM50 subtypes, the Claudin-low subtype was determined by unsupervised hierarchical clustering of all pretreatment and RD posttreatment samples using the intrinsic gene list as described by Parker et al. (53).

TNBCtype-6 subtypes were assigned by submitting normalized and filtered gene expression data, restricted to coding genes with nonzero expression in at least 70% of samples, for all 410 tumors (340 pretreatment, 70 RD) to the TNBCtype online portal (25). Samples failing the ER positivity filter (*n* = 45) were excluded and labeled “Not_Available.”

TNBCtype-4 classifications were derived by remapping TNBCtype-6 calls to the 4 refined subtypes (BL1, BL2, M, LAR) (26). Samples initially classified as IM or MSL were reassigned based on the second-highest centroid correlation, provided it was statistically significant; otherwise, they were labeled “UNS.” For samples labeled UNS in TNBCtype-6, reassignment to a refined subtype was performed if a single subtype showed a positive and statistically significant correlation.

### PDXs and their treatment.

WHIM2 and WHIM30 PDX tumors (54), obtained from Washington University in St. Louis, St. Louis, Missouri, USA, were dissociated and injected (1 × 10^6^ cells in 150 μL of 2.5% FBS HBSS + Matrigel, 1:1) into the right #4 mammary gland of 12-week-old female NSG mice (NOD.Cg-*Prkdc^scid^ Il2rg^tm1Wjl^*/SzJ; The Jackson Laboratory). Once tumors reached 5–7 mm, mice were randomized into treatment groups: untreated, carboplatin/paclitaxel (Carbo/Tax), sacituzumab govitecan (SACI), or trastuzumab deruxtecan (T-DXd). Drug sources were carboplatin — Henry-Schein Medical, paclitaxel — Henry-Schein Medical, SACI (Trodelvy) — UNC Pharmacy Services, and T-DXd (Enhertu) — UNC Pharmacy Services. SACI was suspended in 0.9% saline; T-DXd, molecular-grade water.

Carbo/Tax mice received 50 mg/kg carboplatin + 9 mg/kg paclitaxel weekly for 21 days. SACI groups received 1) 10 mg/kg twice weekly for 21 days, 2) 10 mg/kg weekly, 3) 5 mg/kg twice weekly — both until endpoint or complete regression, or 4) 2.5 mg/kg twice weekly until endpoint. T-DXd mice received 7.5 mg/kg twice weekly for 21 days. All drugs were administered intraperitoneally.

Tumors were measured twice weekly using calipers, and the volume was calculated as V(mm^3^) = (length × width^2^)/2. Monitoring continued until tumors reached 20 mm, tumors ulcerated, 12-week study endpoint, or earlier if body condition score ≤ 2.0 or weight loss ≥ 20%. No treatment group showed consistent weight loss or health decline. All mice were euthanized per institutional guidelines.

### Immunohistochemistry and imaging for PDX tumors.

Immunohistochemistry was performed on untreated WHIM2 and WHIM30 tumors using the anti–estrogen receptor (anti-ER) antibody (Cell Marque, catalog 249R-15-ASR, Clone SP1) at a dilution of 1:30, the anti–progesterone receptor (anti-PR) antibody (Cell Marque, catalog 323R-14-ASR, Clone Y85) at a dilution of 1:30, the anti–human epidermal growth factor receptor 2 (anti-HER2) antibody (Cell Signaling Technology, catalog 4290S, Clone D8F12) at a dilution of 1:800, and the anti–trophoblast cell surface antigen 2 (anti-TROP2) antibody (Enzo, catalog ENZ-ABS380-0100, Clone 01) at a dilution of 1:2,000. Stained tumor sections were whole-slide imaged, and representative images are displayed at 40× original magnification in the figures.

### Data visualization.

Most visualizations — including bar plots, volcano plots, box plots, and scatterplots — were created using the ggplot2 R package (v3.5.2, RRID:SCR_014601). Sankey plots were generated with networkD3 (v0.4.1) and colored in Adobe Illustrator (RRID:SCR_010279). Kaplan-Meier curves were produced using survminer (v0.5.0). GSEA enrichment plots were autogenerated by the GSEA software for Mac (v4.3.3). Venn diagrams were created with the web-based InteractiVenn (55). The graphical abstract was created in BioRender. Raedler, P. (2026) https://BioRender.com/p5h9xgb

### Statistics.

Analyses were conducted using R (v4.4.1) and Python (v3.6). Most statistics were calculated using the stats R package (v.4.4.1, RRID:SCR_025678) unless otherwise noted. For all statistical analyses, *P* values less than 0.05 were considered significant, unless described otherwise. Baseline clinicopathological characteristics were compared using Pearson’s χ^2^ test for categorical variables. Differences in gene expression, signature scores, and DNA amplifications between pretreatment basal-basal and basal-nonbasal tumors as well as differences in tumor volume were assessed using the Wilcoxon test (see Figures 3, 4, and 6, and Supplemental Figures 7–12 and 15). Differences between pretreatment basal-basal tumors and matched posttreatment RD samples were calculated using 2-tailed paired *t* tests, following Shapiro-Wilk tests confirming normality (*P* > 0.05) (Figure 4 and Supplemental Figures 10–12). Data are presented as box plots showing the median, interquartile range (25th–75th percentile), and whiskers extending to the most extreme data point within 1.5 times the interquartile range from the box, with individual data points overlaid as a scatter plot. Tumor growth curves show mean tumor volume; SEM values are not displayed in the figures for visual clarity but are reported in the Supporting Data Values file for time points at which statistical comparisons were performed.

Survival analyses (OS and EFS) used Kaplan-Meier estimates from the survival R package (v2.8-3), with survival probabilities extracted at specified time points (e.g., 5 years). Univariable Cox models were used to assess associations with OS or EFS, reporting HRs, 95% CIs, and *P*s. To assess whether basal-like subtype status improved model fit, Cox proportional hazard models were fit for OS/EFS, first with RCB class alone and then with RCB class and RD basal-like status; a likelihood ratio test was used to compare the fit of the models. Gene signature associations with survival were also evaluated using univariate Cox models, both alone and adjusted for RCB class or its interaction with signature scores. Although FDR correction (Benjamini-Hochberg) was applied, nominal *P* values were reported due to limited adjusted significance. Gene expression differences among tumor subgroups were assessed using 1-way between-subject ANOVA (factor: tumor subgroup, 8 levels), assuming normality and homogeneity of variances; no post hoc tests were performed (Supplemental Figure 17).

To compare the segment-level log_2_ copy number ratios between the basal-basal and basal-nonbasal sample groups, a separate logistic regression model was fit for each copy number segment, predicting sample group membership from the segment log_2_ copy number ratio estimate. Pairwise associations among *CDK6*, *CCND1*, and *CDKN2A/B* were evaluated using a permutation-based global test, summing pairwise χ^2^ statistics and assessing significance via 10,000 column-wise permutations.

### Study approval.

The UNC Office of Human Research Ethics reviewed the correlative science component of this project and concluded that it does not meet the federal definition of human subject research (Study 18-0846). The CALGB 40603 study protocol received approval from the NCI’s central institutional review board (Rockville, Maryland, USA), in addition to the institutional review boards at each participating center. All individuals enrolled in CALGB 40603 gave written informed consent in compliance with applicable federal and institutional requirements. CALGB has since been incorporated into the Alliance for Clinical Trials in Oncology. All animal procedures followed IACUC-approved protocols at the UNC.

### Data availability.

RNA-seq data, DNA-seq data, clinical annotations, and outcome information are publicly accessible through the NCBI database of Genotypes and Phenotypes (dbGaP) phs003801. Underlying data for each figure panel are available in the Supporting Data Values unless the data are protected due to patient privacy regulations, in which case they are available via controlled access in dbGaP. Code to reproduce analyses is available through GitHub (https://github.com/perou-lab/CALGB40603_PrePost_Analyses.git).

## Author contributions

CMP, PDR, and LAC conceptualized the study. PDR and BMF developed the methodology. PDR, BMF, and CMP conducted the investigation and analyzed the data. PDR performed the PDX experiments. PDR and BMF prepared the visualizations. CMP and LAC acquired funding. CMP administered the project and supervised the study. PDR and CMP wrote the original draft of the manuscript. PDR, CMP, LAC, BMF, AFM, ADP, MCH, BS, and WS reviewed and edited the manuscript.

## Conflict of interest

CMP is an equity stockholder and consultant of BioClassifier LLC and Reveal Genomics; CMP is also listed as an inventor on patent applications for the Breast PAM50 Subtyping assay (US Patent No. 12/995,450). WMS is an unpaid member of the steering committee for AbbVie. BS is on the Speakers Bureau for AstraZeneca.

## Funding support

This work is the result of NIH funding, in whole or in part, and is subject to the NIH Public Access Policy. Through acceptance of this federal funding, the NIH has been given a right to make the work publicly available in PubMed Central.

- NIH National Cancer Institute (NCI) U10CA180821 (Alliance for Clinical Trials in Oncology).
- NIH NCI U24CA176171 (Alliance for Clinical Trials in Oncology).
- NIH NCI UG1CA233373 (Alliance for Clinical Trials in Oncology).
- NIH NCI Breast SPORE program P50-CA058223 (CMP).
- Susan G. Komen SAC-160074 (CMP and PDR).
- Breast Cancer Research Foundation BCRF-23-127 (CMP).
- NIH NCI R01-CA229409 (CMP).
- UNC LCCC Triple Negative Breast Cancer Center (CMP).
- Support provided to the Alliance for Clinical Trials in Oncology and Alliance Foundation Trials programs (https://acknowledgments.alliancefound.org).

## Supplementary Material

Supplemental data

ICMJE disclosure forms

Supporting data values

## Version 1 08/04/2026

In-Press Preview

## Version 2 09/15/2026

Electronic publication
