# Supplementary material for: Molecular evaluation of residual disease following neoadjuvant chemotherapy in triple-negative breast cancer CALGB 40603 (Alliance)
Source: J Clin Invest. 2026 Aug 4;136(18):e205962. doi: 10.1172/JCI205962 (PMC13574147; doi:10.1172/JCI205962)
Supplement: Supplementary file 1 [file jci-136-205962-s165.pdf]

Supplemental Figures

Supplemental Figure 1

A

Characteristic	All Pre-Treatment N = 340[1]	Pre-Treatment with matching Post-sample N = 162[1]	p-value[2]
**Tumor Stage, Outcome**			0.6
Stage II, pCR	115 (34%)	53 (33%)	
Stage II, residual disease	116 (34%)	50 (31%)	
Stage III, pCR	54 (16%)	34 (21%)	
Stage III, residual disease	55 (16%)	25 (15%)	
**Subtype (Pre-Treatment)**			>0.9
Basal	263 (77%)	123 (76%)	
Claudin-low	13 (3.8%)	6 (3.7%)	
HER2-enriched	23 (6.8%)	10 (6.2%)	
LuminalA	21 (6.2%)	12 (7.4%)	
LuminalB	2 (0.6%)	2 (1.2%)	
Normal-like	18 (5.3%)	9 (5.6%)	
**Age Group**			0.7
< 40	74 (22%)	33 (20%)	
40-59	208 (61%)	105 (65%)	
>= 60	58 (17%)	24 (15%)	
**Race**			0.9
Asian, Native Hawaiian or Pacific Islander, American Indian or Alaska Native, or More than one race	19 (5.8%)	11 (6.9%)	
Black or African American	65 (20%)	31 (19%)	
White	246 (75%)	118 (74%)	

[1]n (%)

[2]Pearson's Chi-squared test

B

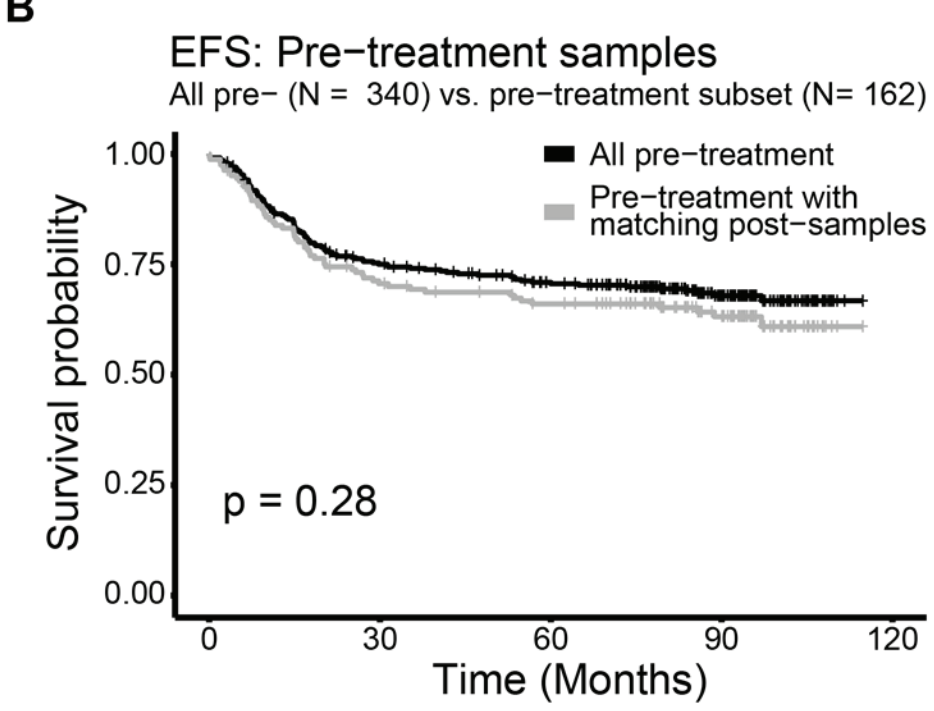

EFS	30 Months[1]	60 Months[1]	90 Months[1]	120 Months[1]
All pre-treatment	75% (71%, 80%)	71% (66%, 76%)	68% (63%, 73%)	67% (61%, 73%)
Pre-treatment with matching post-sample	71% (64%, 78%)	66% (59%, 74%)	63% (56%, 71%)	61% (53%, 70%)

[1]Log-rank test p-value: 0.285

C

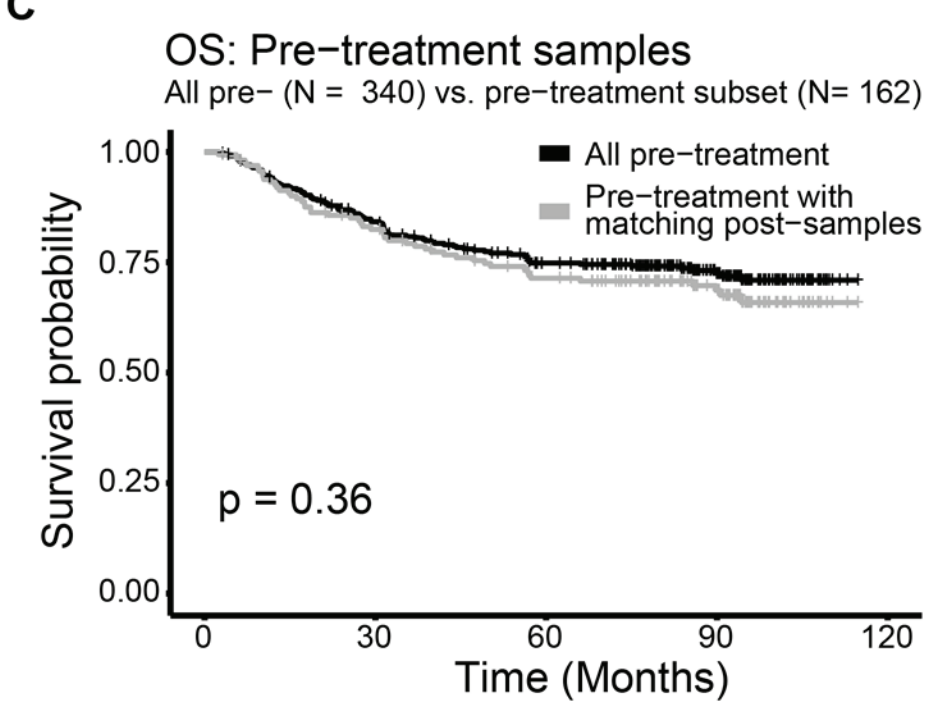

OS	30 Months[1]	60 Months[1]	90 Months[1]	120 Months[1]
All pre-treatment	84% (80%, 88%)	75% (70%, 80%)	72% (68%, 78%)	71% (66%, 77%)
Pre-treatment with matching post-sample	82% (77%, 89%)	71% (65%, 79%)	69% (62%, 77%)	66% (58%, 75%)

[1]Log-rank test p-value: 0.359

Supplemental Figure 1. Comparison of clinical characteristics and survival outcomes in pre-treatment patient cohorts. (**A**) Table comparing patient and tumor characteristics, including tumor stage, pathologic complete response (pCR) status, pre-treatment PAM50 subtype, age, and race, between all pre-treatment patients (N = 340) and the subset with matched post-treatment samples (N = 162). (**B**) Event-free survival (EFS) Kaplan–Meier curves for all pre-treatment patients (black, N = 340) and those with matched post-treatment samples (gray, N = 162). (**C**) Overall survival (OS) Kaplan–Meier curves for the same patient groups as in (B). 5-year survival estimates, number of events, hazard ratios, and associated p-values are presented in Supporting Data.

Supplemental Figure 2

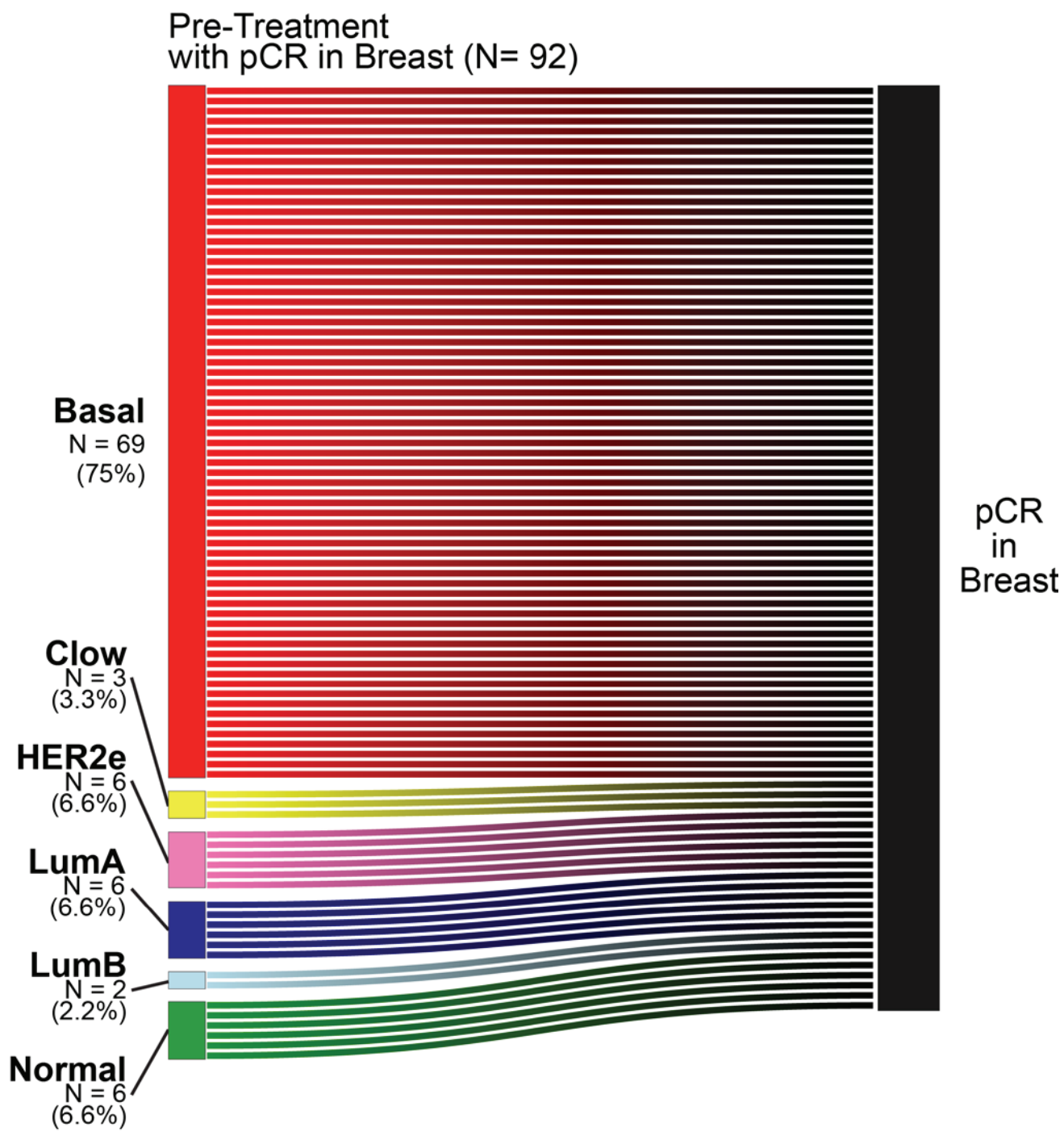

Supplemental Figure 2. PAM50 molecular subtype distribution of pre-treatment specimens from patients that achieved a pCR. Sankey diagram that indicates the PAM50 molecular subtype distribution of 92 pre-treatment specimens, whose patients achieved a pCR in the breast. Subtypes are color-coded as follows: Basal = Basal-like (red), Clow = Claudin-low (yellow), HER2e = HER2-enriched (pink), LumA = Luminal A (dark blue), LumB = Luminal B (light blue), Normal = Normal-like (green), and pCR in the post-treatment is indicated with black.

Supplemental Figure 3

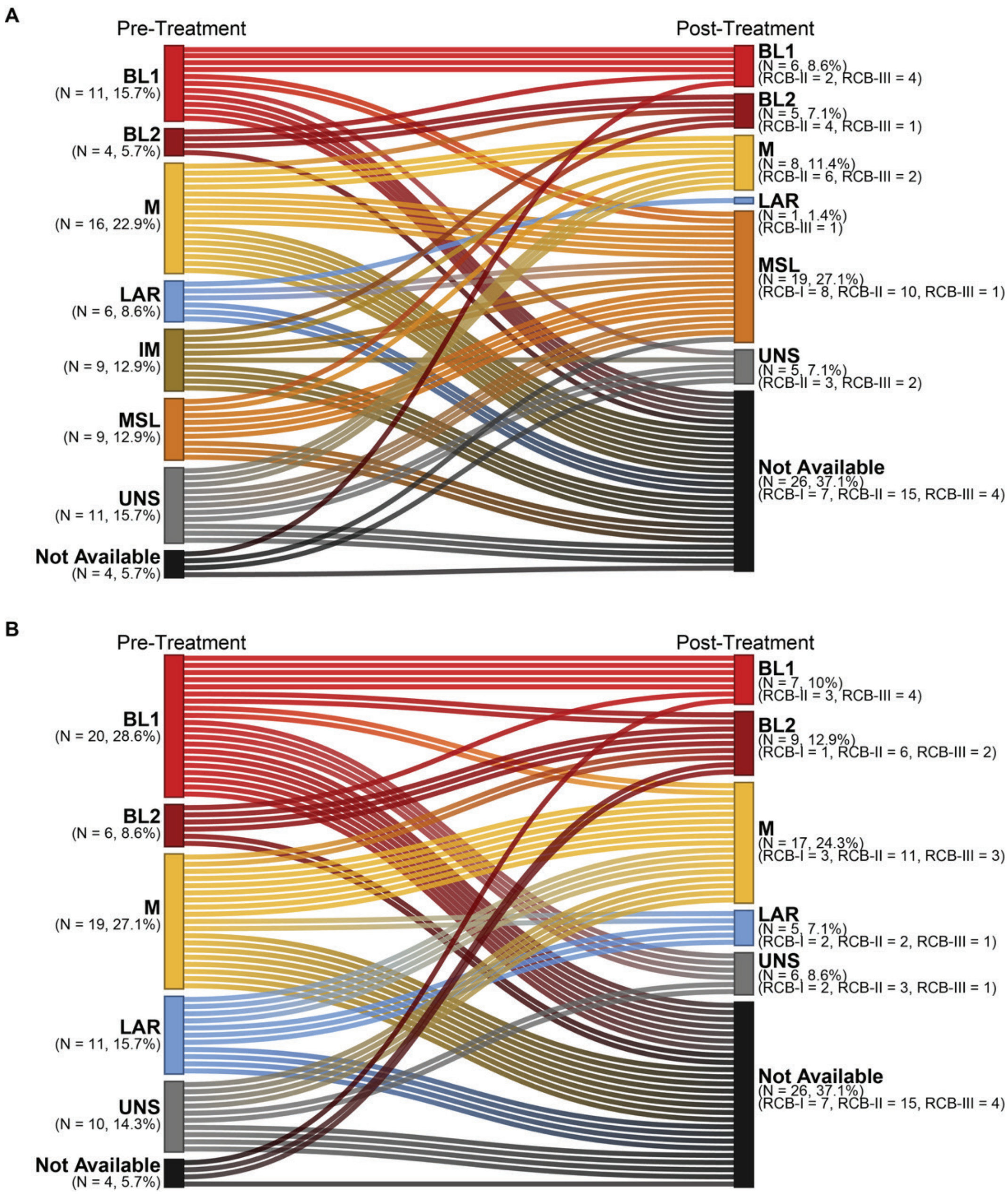

Supplemental Figure 3. Subtypes in matching pre-treatment and post-treatment RD samples using TNBCtype-6 and TNBCtype-4. (**A**) Sankey plot of TNBCtype-6 for pre-treatment tumors and matching post-treatment RD of 70 patients. (**B**) Sankey plot of TNBCtype-4 for pre-treatment tumors and matching post-treatment RD of 70 patients. BL1 = basal-like 1 (red); BL2 = basal-like 2 (dark red); IM = immunomodulatory (gold); M = mesenchymal (yellow); MSL = mesenchymal stem-like (orange); LAR = luminal androgen receptor (blue), UNS = unsure subtype (grey), Not Available = subtype was not determined based on TNBCtype ER+ pre-filtering (black). For each post-treatment subtype the number of samples per RCB class is indicated.

Supplemental Figure 4

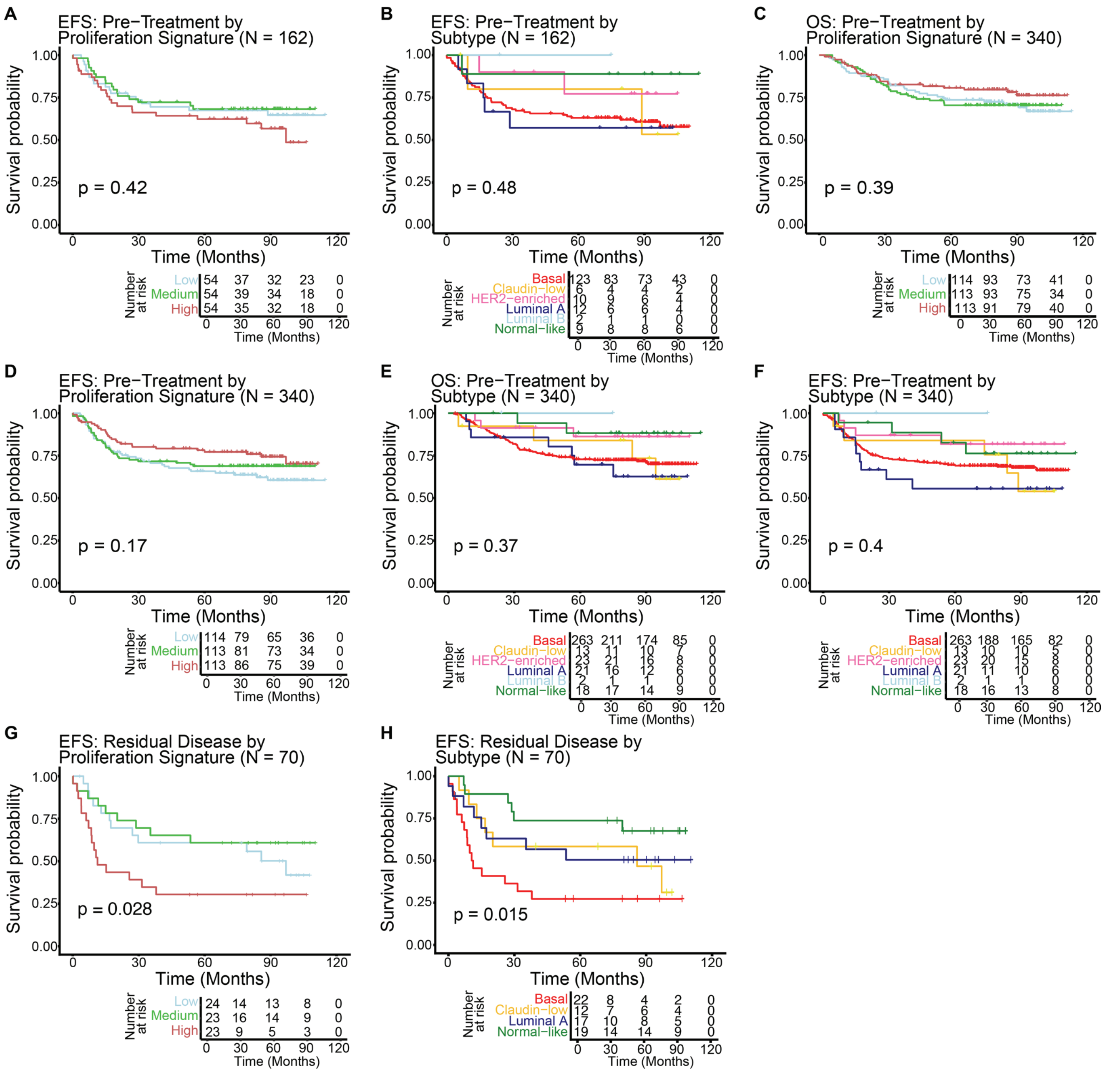

Supplemental Figure 4. Kaplan–Meier curves of EFS and OS stratified by a proliferation gene expression signature and PAM50 molecular breast cancer subtype. EFS was analyzed in pre-treatment tumors with matched post-treatment samples (N = 162) using tertiles of a proliferation signature (GSEA_GP1_Proliferation_DNA_repair.r=0.972_REACTOME_CELL_CYCLE_MITOTIC) (**A**) and molecular subtype (**B**). In all pre-treatment tumors (N = 340), OS and EFS were evaluated by proliferation tertiles (**C and D**, respectively) and by molecular subtype (**E and F**, respectively). Among tumors with RD (N = 70), EFS was assessed by proliferation tertiles (**G**) and by molecular subtype (**H**). Kaplan–Meier plots with proliferation signatures are stratified by tertile, and plots by molecular subtype are color-coded: Basal-like (red), Claudin-low (yellow), Luminal A (dark blue), Luminal B (light blue), and Normal-like (green). All p-values were determined using the log-rank test. 5-year survival estimates, number of events, hazard ratios, and associated p-values are presented in Supporting Data.

Supplemental Figure 5

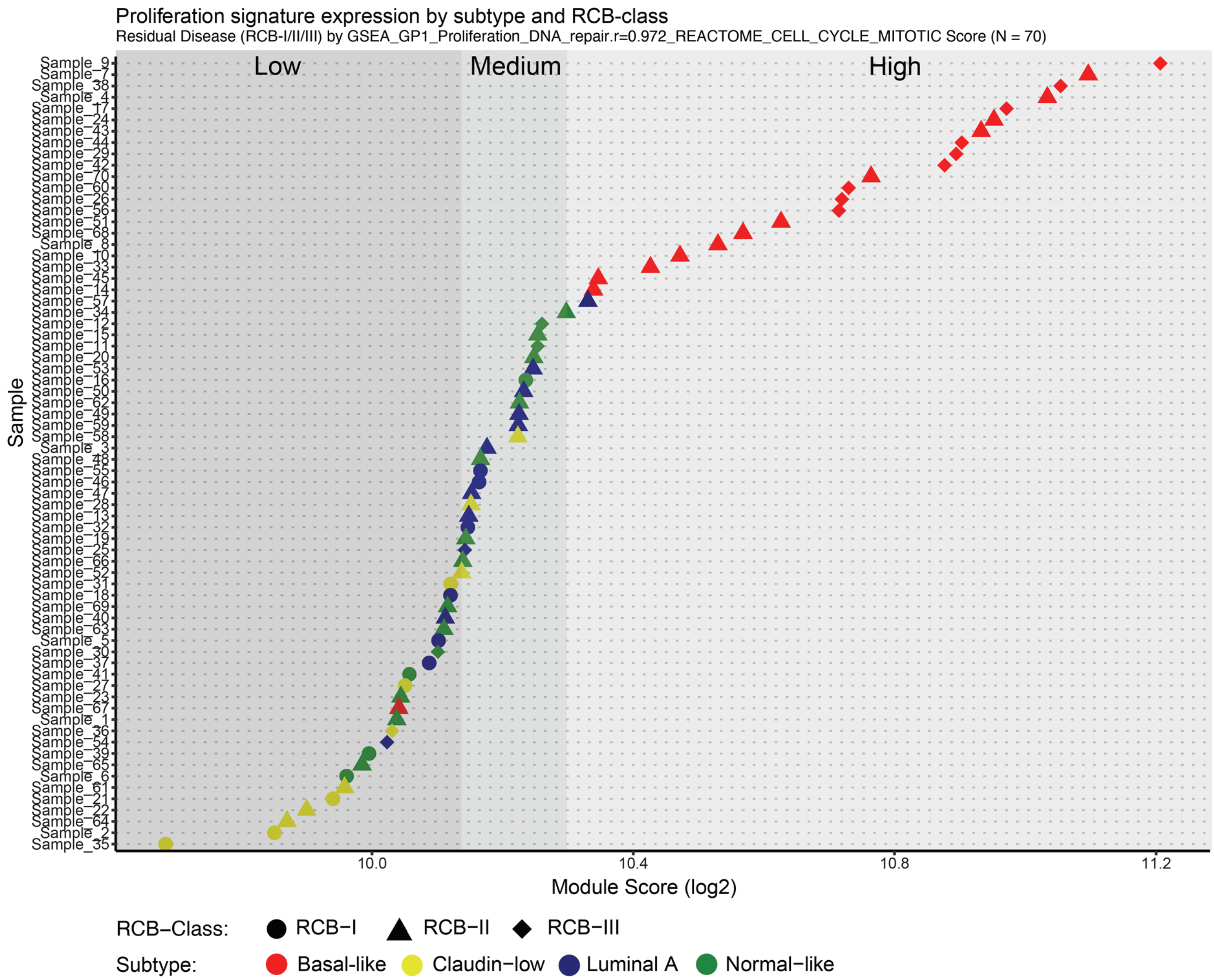

Supplemental Figure 5. Proliferation scores in post-treatment RD samples (N = 70) by PAM50 subtype and RCB class. Each point represents an individual tumor, ordered from lowest to highest proliferation gene expression score. Points are colored by post-treatment RD PAM50 subtype: Basal-like (red), Claudin-low (yellow), Luminal A (dark blue), and Normal-like (green). Point shape indicates RCB class: RCB-I (circle), RCB-II (triangle), and RCB-III (diamond). Background shading denotes the gene expression proliferation tertiles used in Figure 2C: low (dark gray), medium (intermediate gray), and high (light gray) proliferation.

Supplemental Figure 6

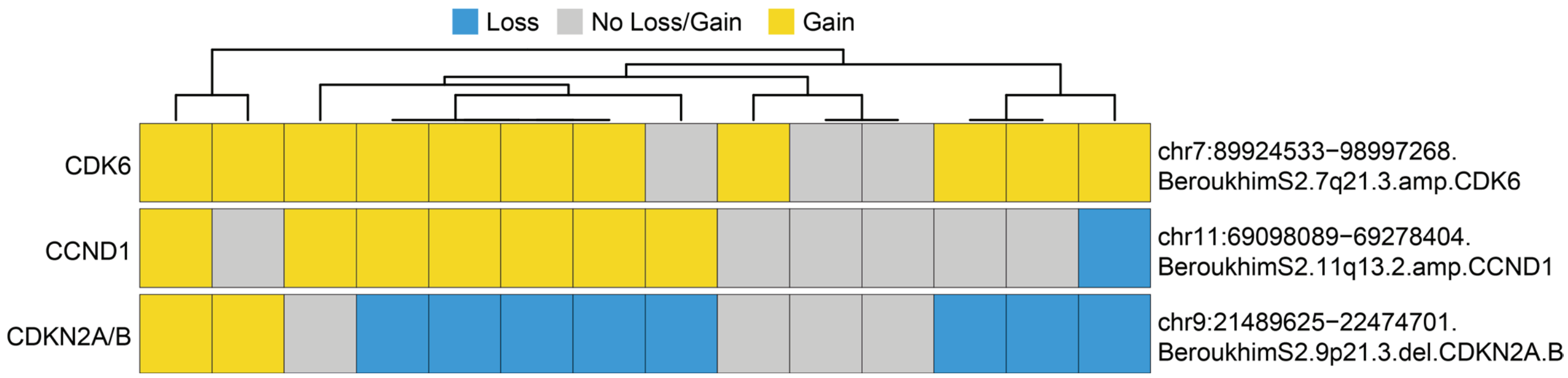

Supplemental Figure 6. Cluster of pre-treatment Basal-Basal DNA copy number gains/losses for *CDK6*, *CCND1*, and *CDKN2A/B*. Hierarchical cluster of pre-treatment Basal-Basal tumors (N = 14) using DNA gain (yellow), loss (blue), and no loss/gain (grey) for *CDK6*, *CCND1*, and *CDKN2A/B*. Chromosomal locations are indicated on the right y-axis. Underlying data is available in the Supporting Data.

Supplemental Figure 7

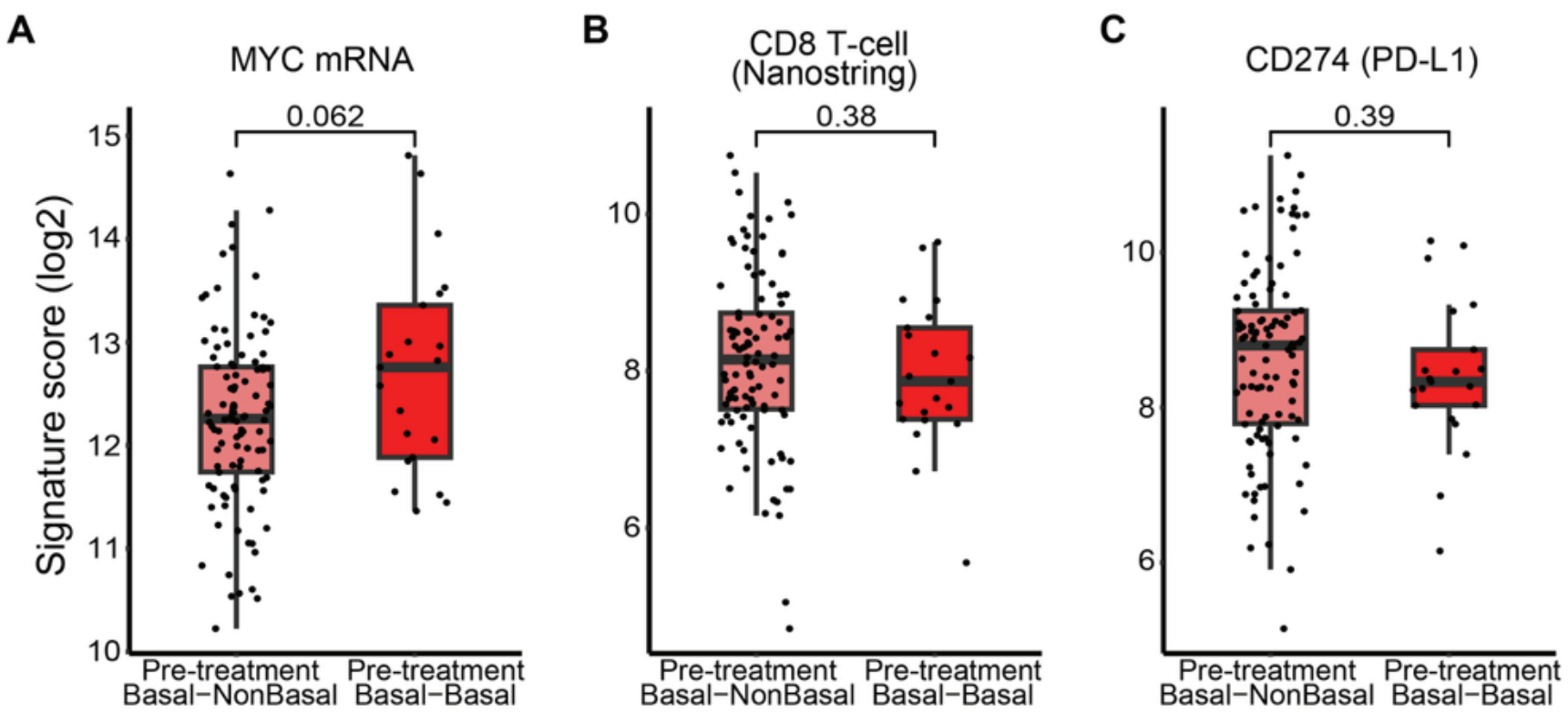

Supplemental Figure 7. Gene expression differences between pre-treatment Basal-Basal and pre-treatment Basal-NonBasal samples. Box plots comparing log2 signature score of (**A**) *MYC* mRNA, (**B**) CD8 T-cell signature, and (**C**) *CD274* mRNA between pre-treatment Basal-Basal (red) and pre-treatment Basal-NonBasal (light red) samples. For all box plots individual data points are shown, and p-values were calculated using the Wilcoxon rank-sum test.

Supplemental Figure 8

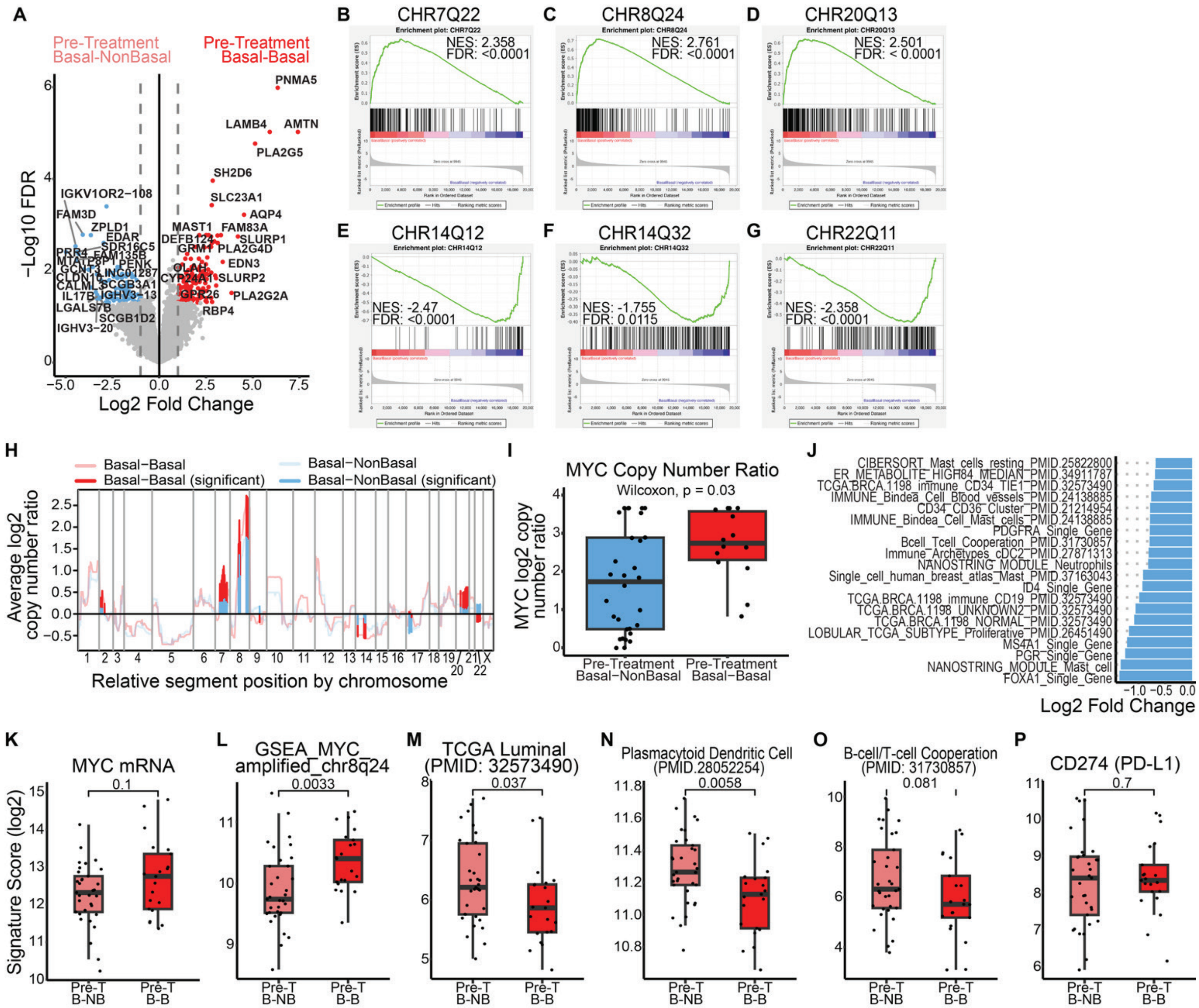

Supplemental Figure 8. Comparative molecular profiling between Basal-NonBasal and Basal-Basal pre-treatment tumors of patients with RD. (**A**) Volcano plot illustrating differential gene expression between Basal-NonBasal (N = 33) and Basal-Basal (N = 21) samples; genes significantly upregulated in Basal-Basal (FDR < 0.05, $log_2FC > 1$) are highlighted in red, while those downregulated (FDR < 0.05, $log_2FC < -1$) are shown in blue. (**B-G**) Gene Set Enrichment Analysis (GSEA) plots displaying chromosomal segments with significant enrichment in Basal-Basal; (**B-D**) positively enriched regions (Chr7q22, Chr8q24, Chr20q13), and (**E-G**) negatively enriched regions (Chr14q12, Chr14q32, Chr22q11). (**H**) Bar plot representing the average log_2 copy number ratio across samples; bold red bars indicate regions significantly (p-value < 0.05) amplified/deleted in Basal-Basal, whereas bold blue bars denote regions significantly amplified/deleted in Basal-NonBasal. (**I**) Box plot of log_2-transformed *MYC* copy number ratios comparing Basal-NonBasal (blue) and Basal-Basal (red). (**J**) Bar plot of gene signatures differentially expressed between Basal-NonBasal and Basal-Basal (q-value < 0.05); signatures downregulated are displayed in blue. (**K**, **L**) Box plots displaying *MYC* gene expression levels for pre-treatment Basal-NonBasal (Pre-T B-NB, light red) and pre-treatment Basal-Basal (Pre-T B-B, red) (**K**), alongside gene signature expression for the *MYC*-amplified region Chr8q24 (**L**). (**M-P**) Box plots comparing expression of the TCGA Luminal (**M**), plasmacytoid dendritic cells (**N**), and B-cell/T-cell cooperation (**O**) gene expression signatures, as well as *CD274* gene expression (**P**) between pre-treatment Basal-NonBasal and pre-treatment Basal-Basal. For all box plots individual data points are shown, and p-values were calculated using the Wilcoxon rank-sum test.

Supplemental Figure 9

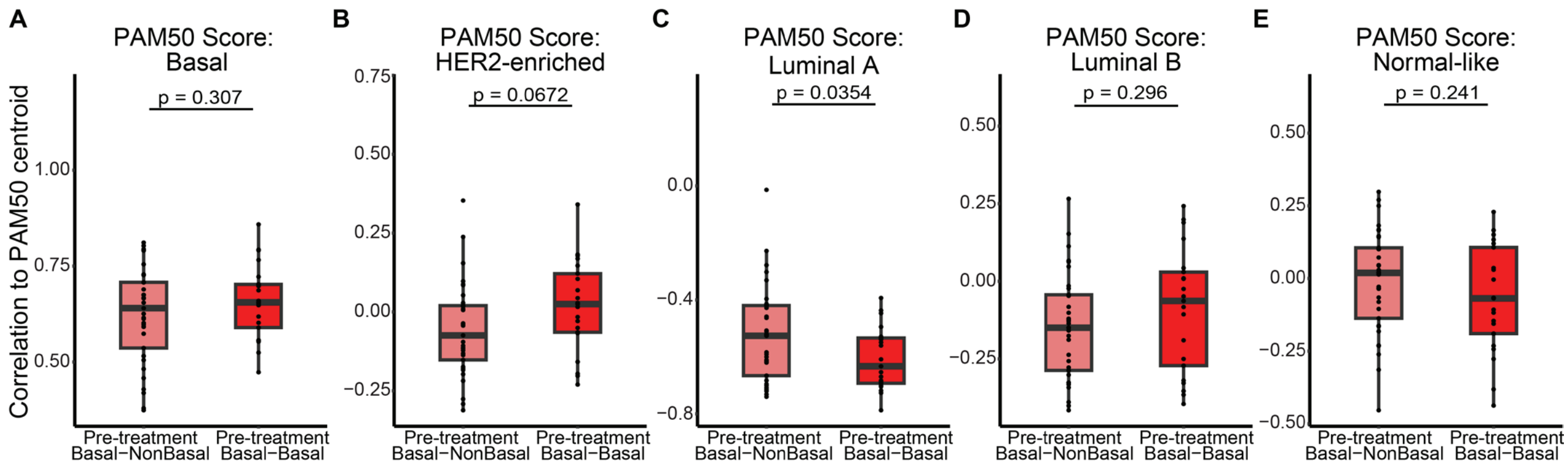

Supplemental Figure 9. PAM50 subtype correlation scores in pre-treatment Basal-NonBasal and pre-treatment Basal-Basal samples. Box plots display scores, which represent the correlation to the centroid to each PAM50 subtype: (**A**) Basal-like, (**B**) HER2-enriched, (**C**) Luminal A, (**D**) Luminal B, and (**E**) Normal-like. Light red indicates pre-treatment Basal-like tumors that did not remain Basal-like post-treatment (Basal-NonBasal, N = 102), and red indicates pre-treatment Basal-like tumors that remained Basal-like post-treatment (Basal-Basal, N = 21). For all box plots individual data points are shown, and p-values were calculated using the Wilcoxon rank-sum test.

Supplemental Figure 10

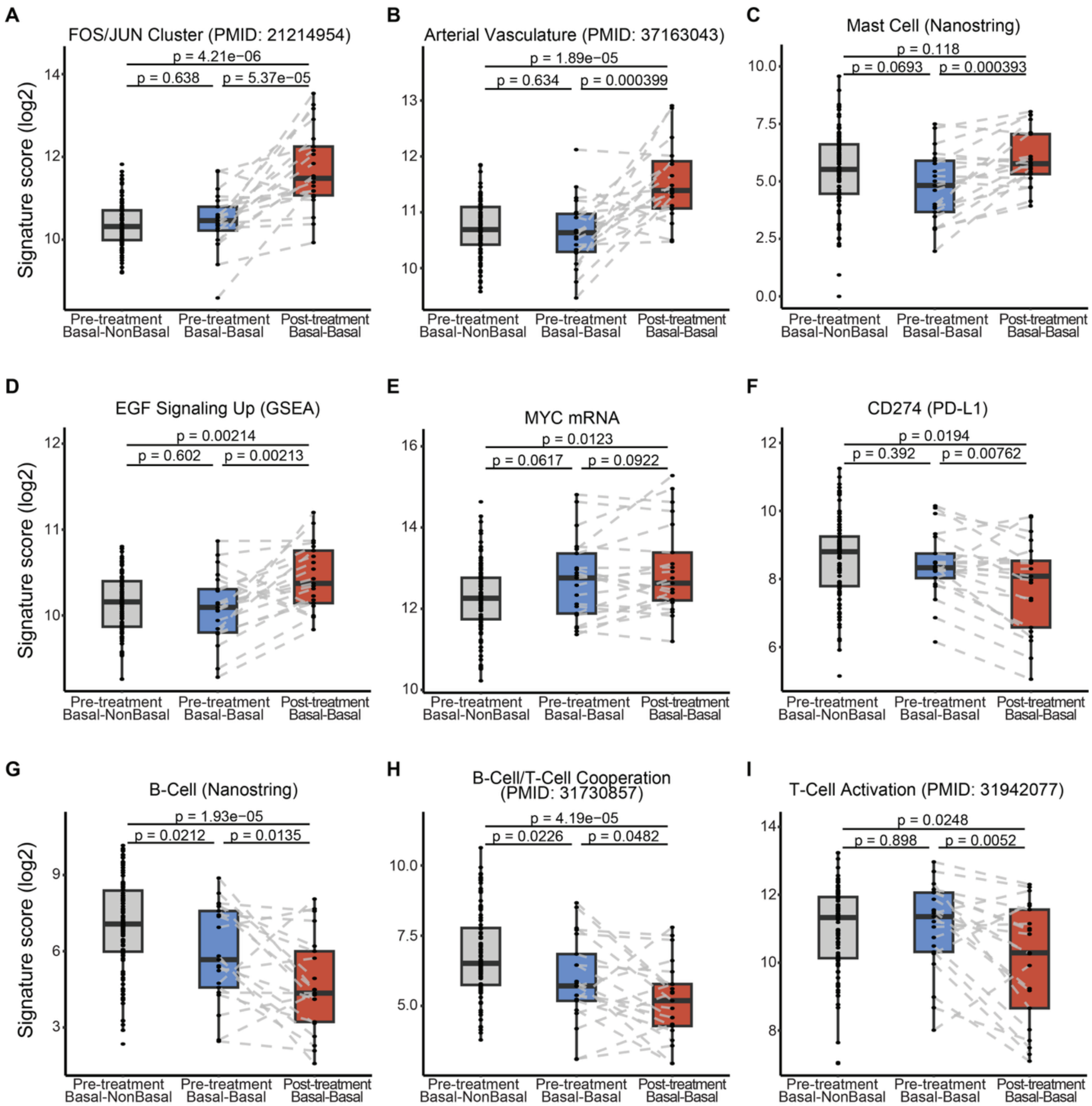

Supplemental Figure 10. Transcriptomic changes in matched pre-treatment Basal-like tumors and post-treatment Basal-like RD. Box plots of log2 signature scores of FOS/JUN Cluster signature (**A**), Arterial Vasculature signature (**B**), Mast Cell signature (**C**), EGF Signaling Up signature (**D**), *MYC* mRNA (**E**), *CD274* mRNA (**F**), B-cell signature (**G**), B-cell/T-cell Cooperation signature (**H**), and T-cell Activation signature (**I**). For box plots individual data points are shown, and p-values were determined using Wilcoxon rank-sum tests for pre-treatment Basal-NonBasal (grey, N = 102) vs. pre-treatment Basal-Basal (blue, N = 21) and pre-treatment Basal-NonBasal vs. post-treatment Basal-Basal (red, N = 21) groups, and paired t-tests for pre-treatment Basal-Basal vs. their matched post-treatment samples.

Supplemental Figure 11

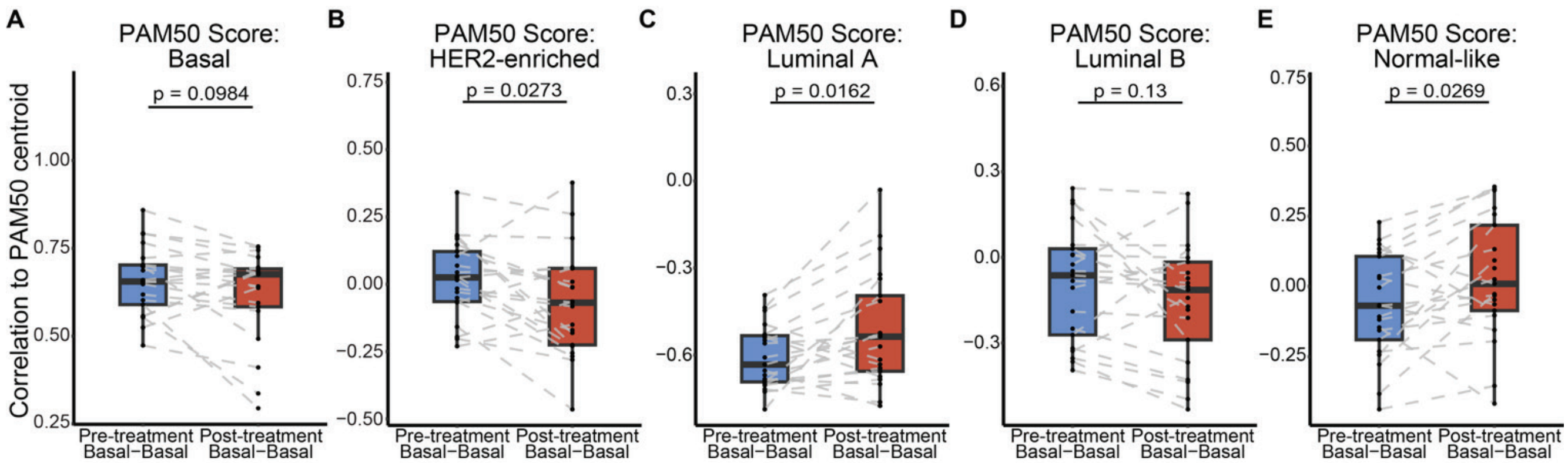

Supplemental Figure 11. PAM50 subtype correlation scores in matched pre- and post-treatment Basal-like tumors (N = 21 pairs). Box plots display scores, which represent the correlation to the centroid to each PAM50 subtype: (**A**) Basal-like, (**B**) HER2-enriched, (**C**) Luminal A, (**D**) Luminal B, and (**E**) Normal-like. Blue indicates pre-treatment Basal-like tumors that remained Basal-like post-treatment (pre-treatment Basal-Basal, N = 21), and red indicates the corresponding post-treatment residual disease samples (post-treatment Basal-Basal, N = 21). For all box plots individual data points are shown, and p-values were calculated using a paired t-test.

Supplemental Figure 12

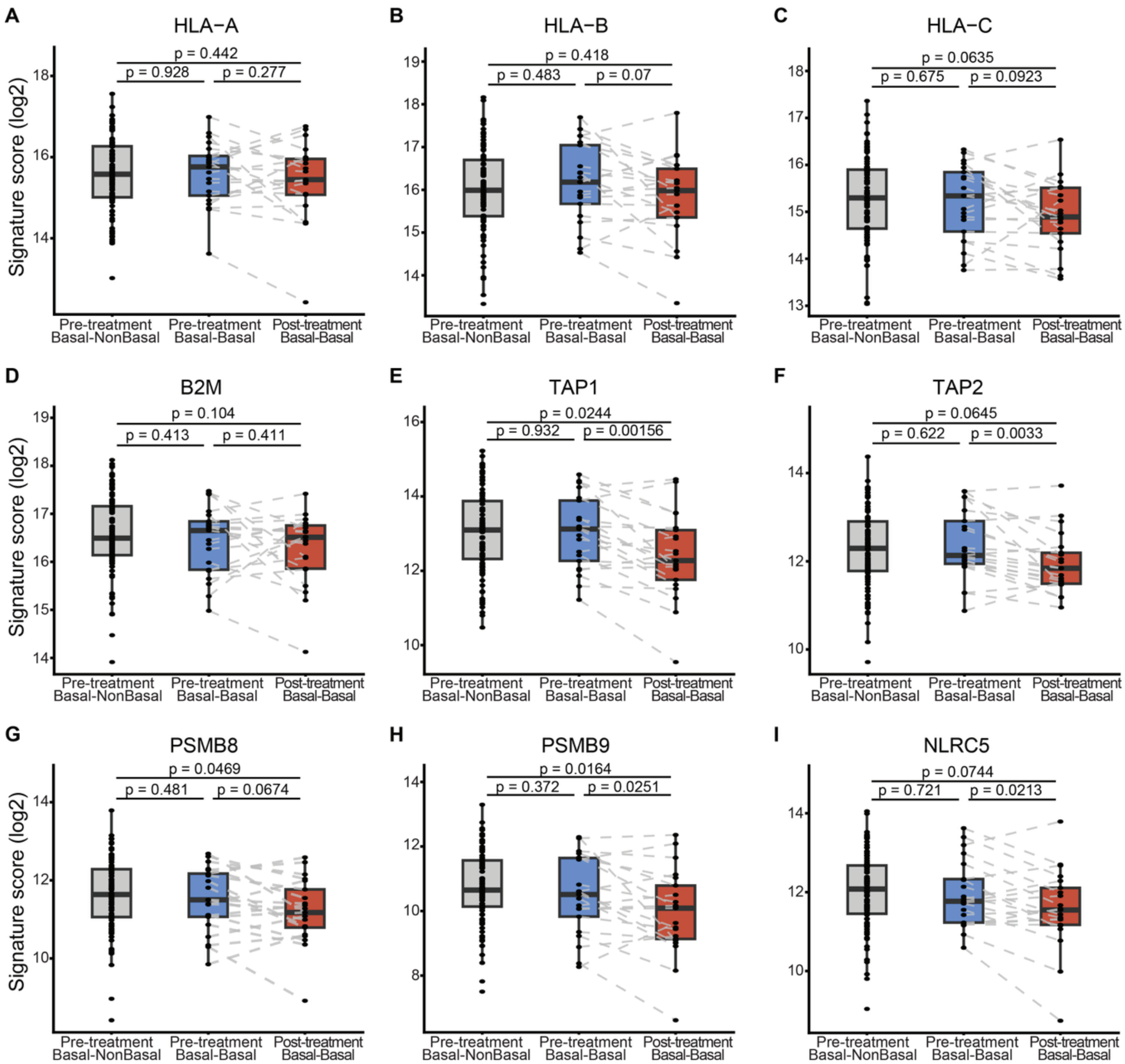

Supplemental Figure 12. Individual gene expression of the MHC Class I signature in matched pre- and post-treatment Basal-like tumors (N = 21 pairs). Box plots display log2 gene expression of the individual genes that comprise the MHC Class I signature displayed in Figure 4L. (**A-C**) Antigen presentation–related genes *HLA-A*, *HLA-B*, and *HLA-C* (encoding MHC Class I molecules). (**D**) *B2M*, encoding a protein that stabilizes MHC Class I complexes. (**E, F**) Genes *TAP1* and *TAP2*, which encode transporters associated with antigen processing. (**G, H**) *PSMB8* and *PSMB9*, encoding catalytic subunits of the immunoproteasome. (**I**) *NLRC5*, encoding a transcriptional regulator of MHC Class I gene expression. Samples are color coded as follows: pre-treatment Basal-NonBasal (grey, N = 102), pre-treatment Basal-Basal (blue, N = 21), and matching post-treatment Basal-Basal (red, N = 21). For all box plots individual data points are shown, and p-values were calculated using unpaired Wilcoxon t-tests, except for the paired pre/post-treatment Basal-Basal samples where a paired t-test was used.

Supplemental Figure 13

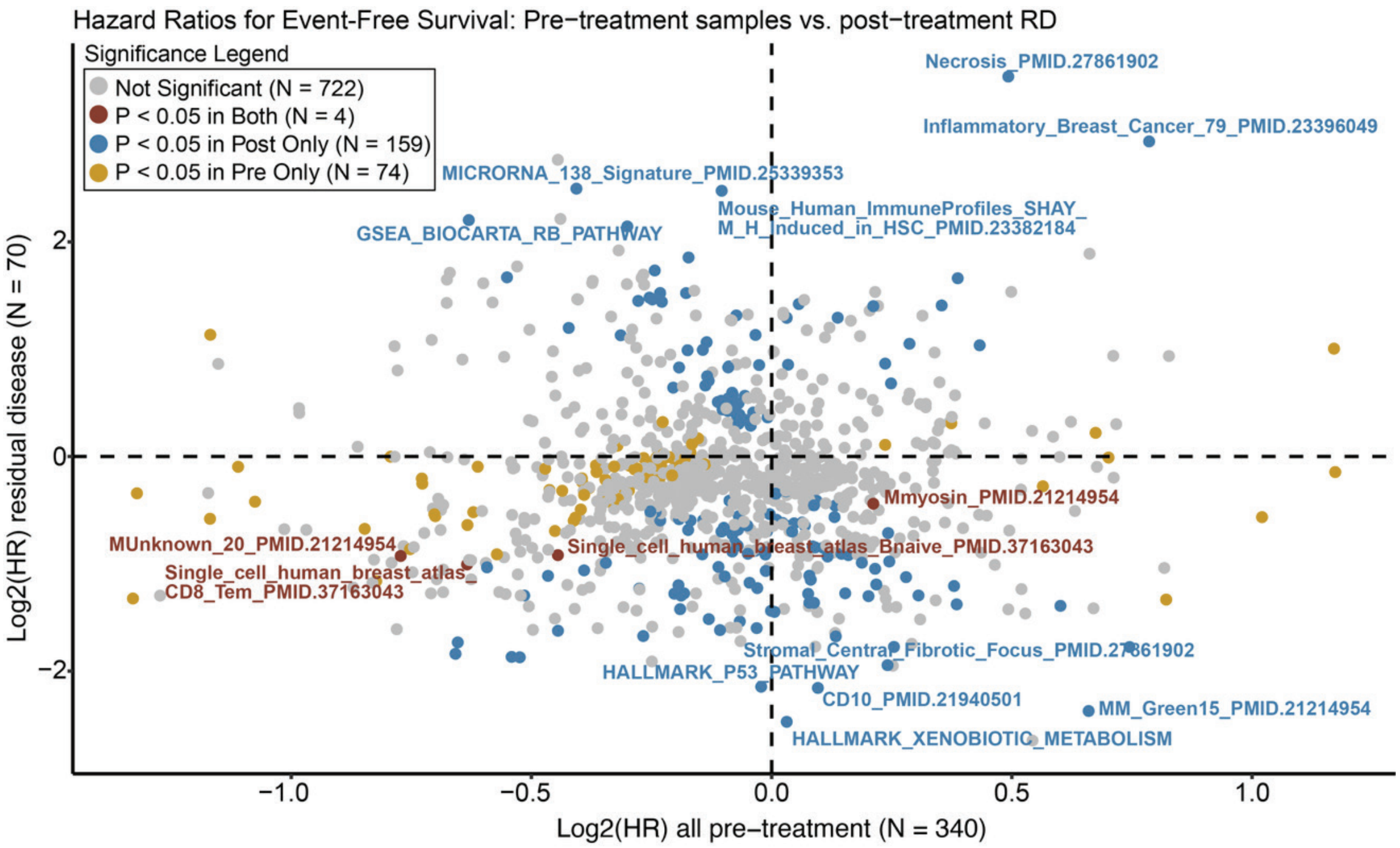

Supplemental Figure 13. Comparison of cox model HRs for EFS in pre-treatment samples and post-treatment RD using gene expression signatures (N = 959). HRs for all pre-treatment tumors (N = 340) versus RD samples (N = 70). HRs are derived from cox proportional hazard modeling using gene signature expression as continuous covariate. Pre-treatment HRs are plotted on the x-axis, and RD HRs on the y-axis. Colored points indicate gene signatures significantly associated with EFS ($p < 0.05$): maroon denotes signatures significant in both pre-treatment and RD samples, yellow indicates significance in pre-treatment only, and blue indicates significance in RD only. Axes display log2-transformed HRs.

Supplemental Figure 14

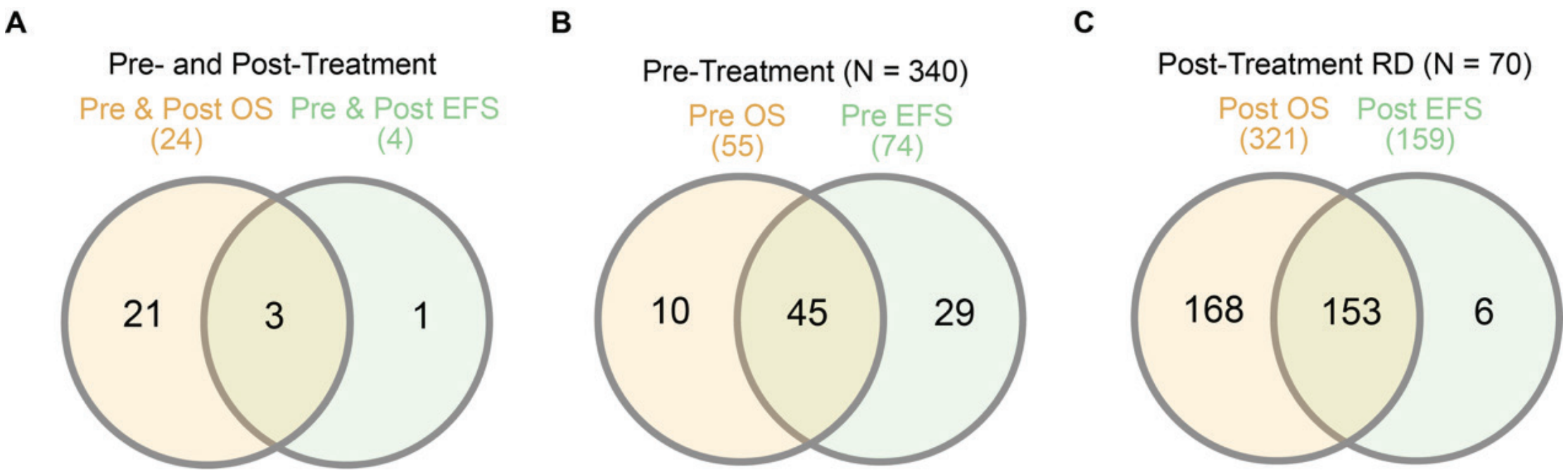

Supplemental Figure 14. Venn diagrams illustrating the overlap of gene expression signatures significantly associated with OS and EFS across different treatment timepoints. (**A**) Signatures significantly associated with both OS and EFS in the full pre-treatment cohort (N = 340) and in the post-treatment RD cohort (N = 70). (**B**) Signatures significant for both OS and EFS in the pre-treatment cohort only (N = 340). (**C**) Signatures significant for both OS and EFS in the post-treatment RD cohort only (N = 70). These analyses include both shared and distinct prognostic gene expression signatures.

Supplemental Figure 15

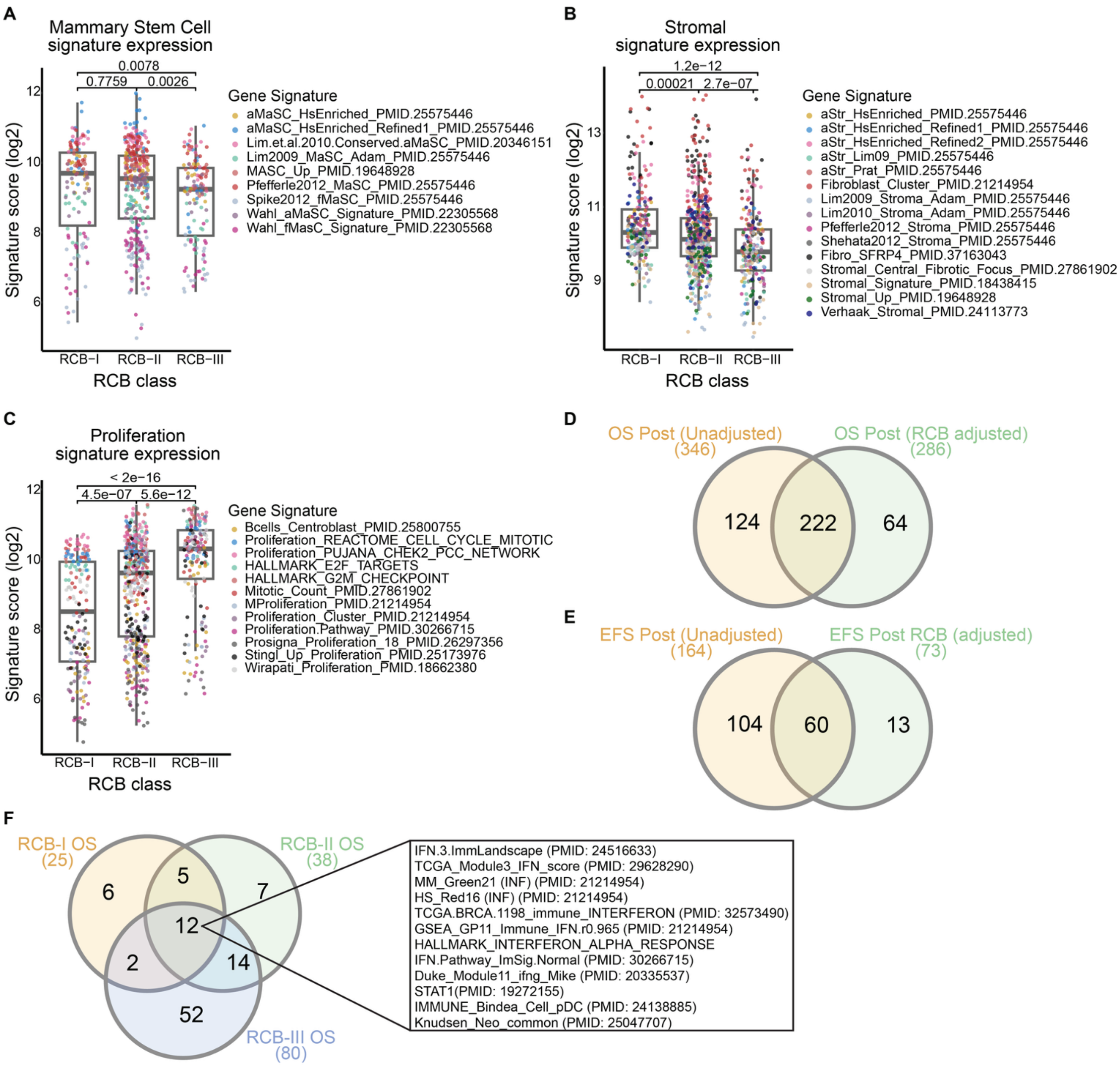

Supplemental Figure 15. Gene expression signatures associated with survival outcomes across RCB classes in post-treatment residual disease (RD, N = 70). (**A-C**) Box plots of nine mammary stem cell signatures (**A**), fifteen stromal signatures (**B**), and twelve proliferation-associated signatures (**C**) across RCB-I, RCB-II, and RCB-III within RD samples (N = 70), each plotted in a different color; all signatures shown were significantly associated with OS or EFS. For all box plots individual data points are shown and p-values were determined by Wilcoxon t-tests. (**D**) Venn diagram of OS-associated signatures from univariate and RCB-adjusted multivariate Cox models. (**E**) Venn diagram of EFS-associated signatures from univariate and RCB-adjusted multivariate Cox models. (**F**) Venn diagram of OS-associated signatures from a Cox model including RCB class as an interaction term, showing shared and class-specific prognostic signatures across RCB-I (N = 15), RCB-II (N = 40), and RCB-III (N = 15); the 12 signatures shared across all RCB classes are highlighted in the adjacent box.

Supplemental Figure 16

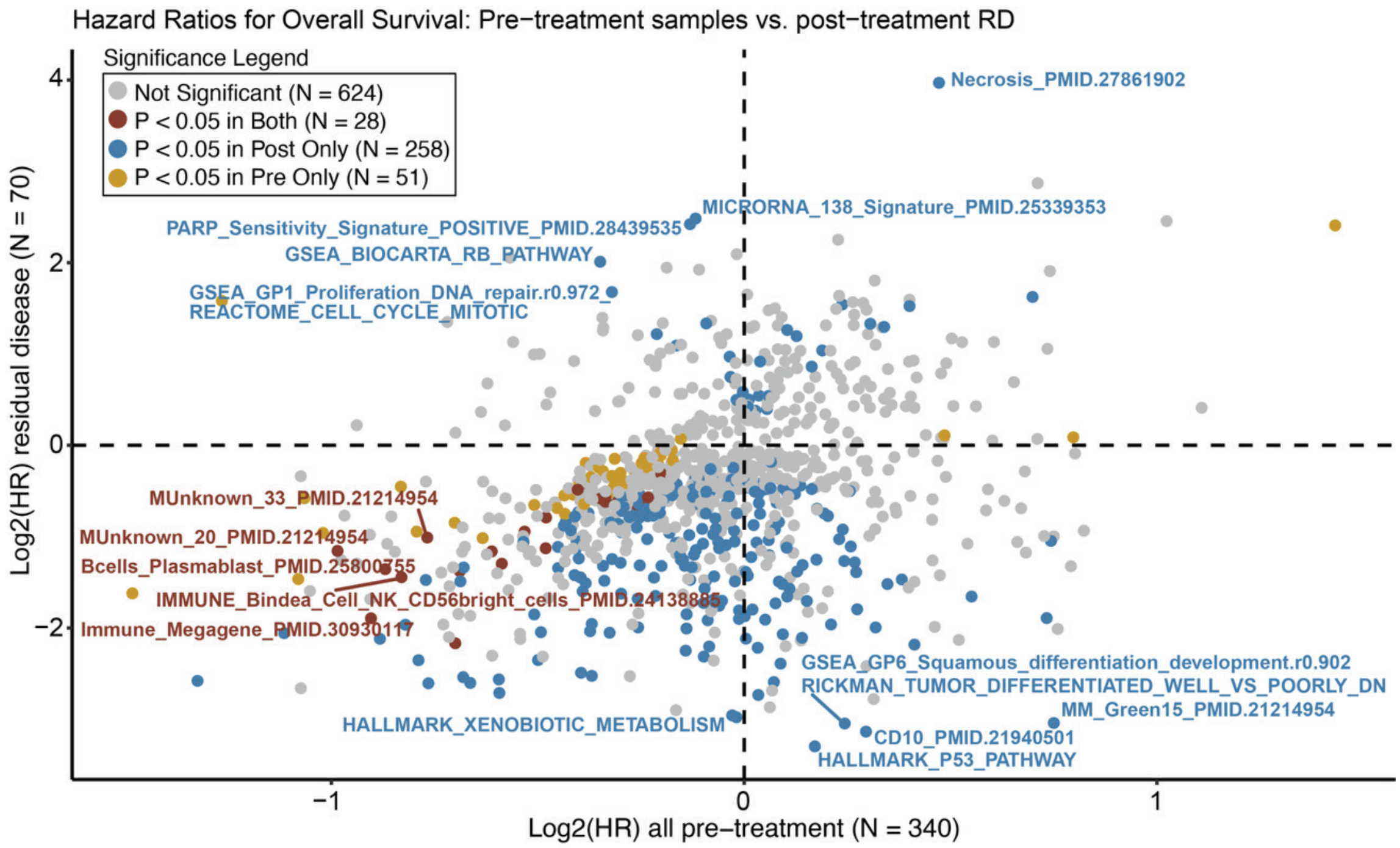

Supplemental Figure 16. Comparison of cox model hazard ratios for OS in pre-treatment samples and RCB-adjusted post-treatment RD samples using gene expression signatures (N = 959). HRs for all pre-treatment tumors (N = 340) versus RCB-adjusted RD samples (N = 70). HRs are derived from cox proportional hazard modeling using gene signature expression as continuous covariate. Unadjusted pre-treatment HRs are plotted on the x-axis, and RCB-adjusted RD HRs are on the y-axis. Colored points indicate gene signatures significantly associated with OS ($p < 0.05$): maroon denotes signatures significant in both pre-treatment and RCB-adjusted RD samples, yellow indicates significance in pre-treatment only, and blue indicates significance in RCB-adjusted RD only. Axes display log2-transformed HRs.

Supplemental Figure 17

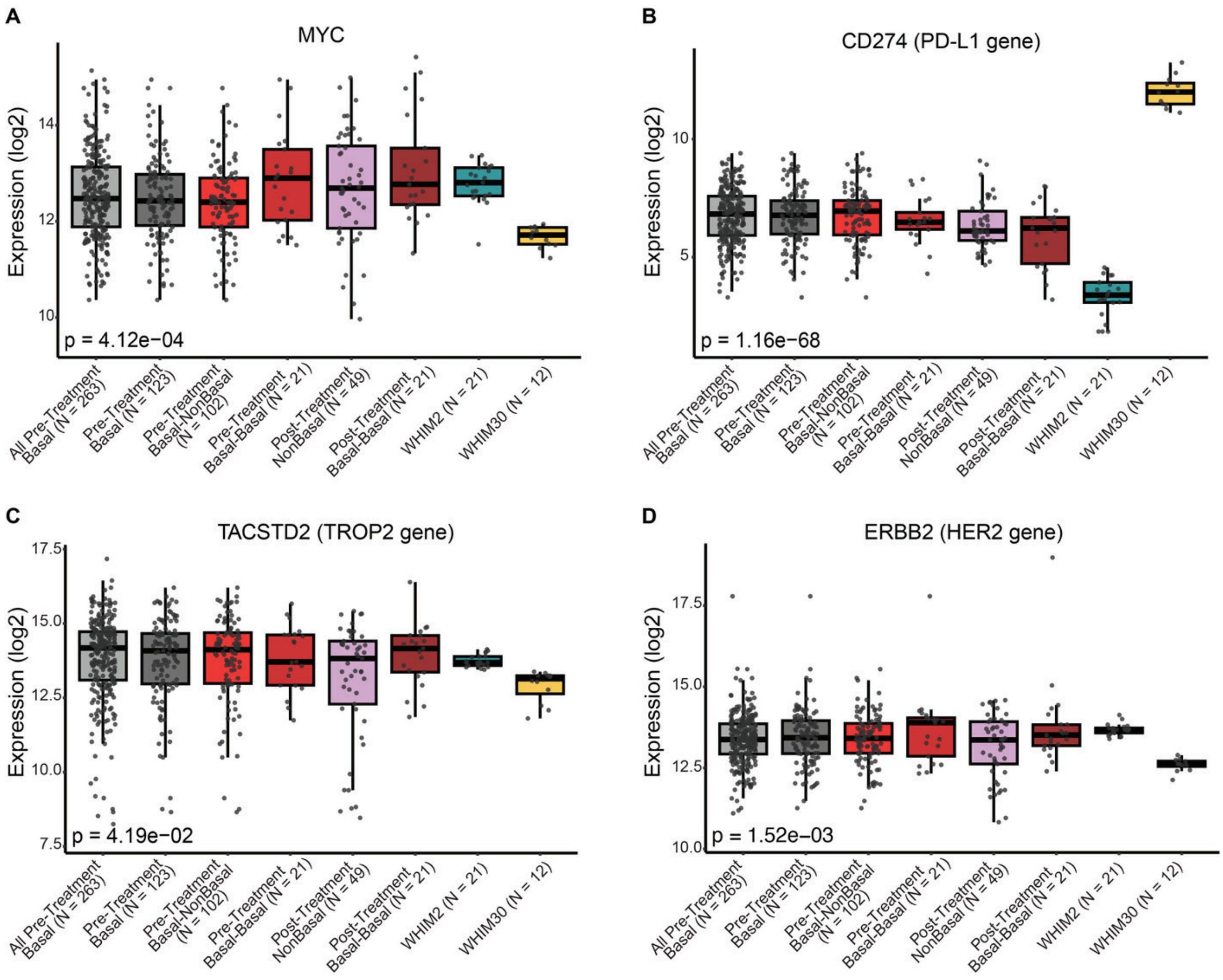

Supplemental Figure 17. Gene expression of selected genes in WHIM models compared to Basal-like TNBC samples from CALGB 40603. Box plots show expression levels of (**A**) *MYC*, (**B**) *CD274* (PD-L1), (**C**) *TACSTD2* (TROP2; sacituzumab govitecan target), and (**D**) *ERBB2* (HER2; trastuzumab deruxtecan target) across integrated, batch-adjusted datasets including CALGB 40603 and PDX tumors WHIM2 and WHIM30. Groups, ordered left to right, include all pre-treatment Basal-like TNBCs (light gray, N = 263), Basal-like pre-treatment TNBCs with matched post-treatment samples (dark gray, N = 123), pre-treatment Basal-NonBasal (light red, N = 102), pre-treatment Basal-Basal (red, N = 21), post-treatment NonBasal (lavender, N = 49), post-treatment Basal-Basal RD (dark red, N = 21), and Basal-like PDX tumors WHIM2 (turquois, N = 21) and WHIM30 (yellow, N = 12). Box plots show gene expression in WHIM tumors relative to clinical Basal-like TNBCs across treatment timepoints. For all box plots individual data points are shown, and p-values were calculated using ANOVA.
